# Hi-C Chromatin Interaction Networks Predict Co-expression in the Mouse Cortex

**DOI:** 10.1371/journal.pcbi.1004221

**Published:** 2015-05-12

**Authors:** Sepideh Babaei, Ahmed Mahfouz, Marc Hulsman, Boudewijn P. F. Lelieveldt, Jeroen de Ridder, Marcel Reinders

**Affiliations:** 1 Delft Bioinformatics Lab, Delft University of Technology, Delft, The Netherlands; 2 Division of Image Processing, Department of Radiology, Leiden University Medical Center, Leiden, The Netherlands; 3 Department of Clinical Genetics, VU University Medical Center, Amsterdam, The Netherlands; 4 Department of Intelligent Systems, Delft University of Technology, Delft, The Netherlands; Centre de Regulacio Genomica, SPAIN

## Abstract

The three dimensional conformation of the genome in the cell nucleus influences important biological processes such as gene expression regulation. Recent studies have shown a strong correlation between chromatin interactions and gene co-expression. However, predicting gene co-expression from frequent long-range chromatin interactions remains challenging. We address this by characterizing the topology of the cortical chromatin interaction network using scale-aware topological measures. We demonstrate that based on these characterizations it is possible to accurately predict spatial co-expression between genes in the mouse cortex. Consistent with previous findings, we find that the chromatin interaction profile of a gene-pair is a good predictor of their spatial co-expression. However, the accuracy of the prediction can be substantially improved when chromatin interactions are described using scale-aware topological measures of the multi-resolution chromatin interaction network. We conclude that, for co-expression prediction, it is necessary to take into account different levels of chromatin interactions ranging from direct interaction between genes (i.e. small-scale) to chromatin compartment interactions (i.e. large-scale).

## Author Summary

Regulatory elements can target genes over large genomic distances through long-range chromatin interactions. These interactions arise as a result of the three-dimensional (3D) conformation of chromosomes in the cell nucleus. This 3D conformation can also result in the co-localization of co-regulated genes. To investigate this, we asked whether genome-wide chromatin interactions can predict co-expression patterns of genes. To address this question, we characterized 3D interactions between genes, captured by Hi-C measurements, by a network, termed chromatin interaction network (CIN). We applied scale-aware topological measures to the network to comprehensively characterize the chromatin interactions at different scales, ranging from direct interaction between gene pairs to chromatin compartment interactions. We then used multi-scale chromatin interactions to predict spatial co-expression patterns in the mouse cortex. The results show that the prediction performance improves when scale-aware topological measures of the multi-resolution chromatin interaction network are used.

## Introduction

The three dimensional (3D) conformation of chromosomes in the cell nucleus plays an important role in determining which genes are expressed in a cell [1–6]. In particular, it has been shown that genes are often regulated by elements that are located far away in terms of the linear genome sequence [7, 8]. In fact, transcribed genes tend to spatially associate with their regulatory elements which results in 3D clustering of co-regulated genes [7, 9]. Moreover, there is increasing evidence that transcription occurs at specific nuclear sites, sometimes called transcription factories [7, 10].

Chromosome conformation capture techniques, such as 3C, 4C, 5C, and Hi-C, allow direct measurement of chromatin interactions and thereby the study of the role of these interactions in gene regulation [11–13]. Using 4C, for instance, it was demonstrated that the 3D structure of the yeast genome correlates with gene co-expression [3]. Dong et al. [2] used Hi-C data from two human cell lines to demonstrate that chromatin interactions associate with co-expression [2]. Both studies, however, have shown that it is difficult to explain the relationship between co-expression and the 3D structure of the genome by considering direct chromatin interactions only. Thus, while a clear relation between chromatin interaction and co-expression exists [2–4], this relation may be better understood if more comprehensive characterizations of long-range chromatin interactions, i.e. those involving also indirect interactions, are taken into account [14].

A more comprehensive characterization of long-range chromatin interactions can be obtained by considering the chromatin conformation data as a network [15, 16]. In such network, termed Chromatin Interaction Network (CIN), a genomic locus is represented by a node while links between the nodes denote chromatin interactions. Investigation of the CIN topology may reveal properties of the 3D genome organization that are important for understanding its function, such as co-expression of genes.

Characterizing the topology in biological networks has been extensively explored, for instance to gain insight into the functional relationships encoded in such networks [17, 18]. Standard network topological measures, such as shortest path, betweenness centrality and clustering coefficient, have been used to capture either the topology around a single node or the global topology of the whole network [19, 20]. As a result, these measures of network topology operate at a fixed zoom-level. Recently, scale-aware topological measures have been shown to superiorly predict gene function and interactions by characterizing the topology of protein interaction networks at different scales [18, 21]. In this work, we explore the use of scale-aware topological measures (STMs), proposed in [21], to describe the CIN topology. Analyzing the CIN topology enables us to study the relation between long-range chromatin interactions and co-expression.

**Fig 1:**
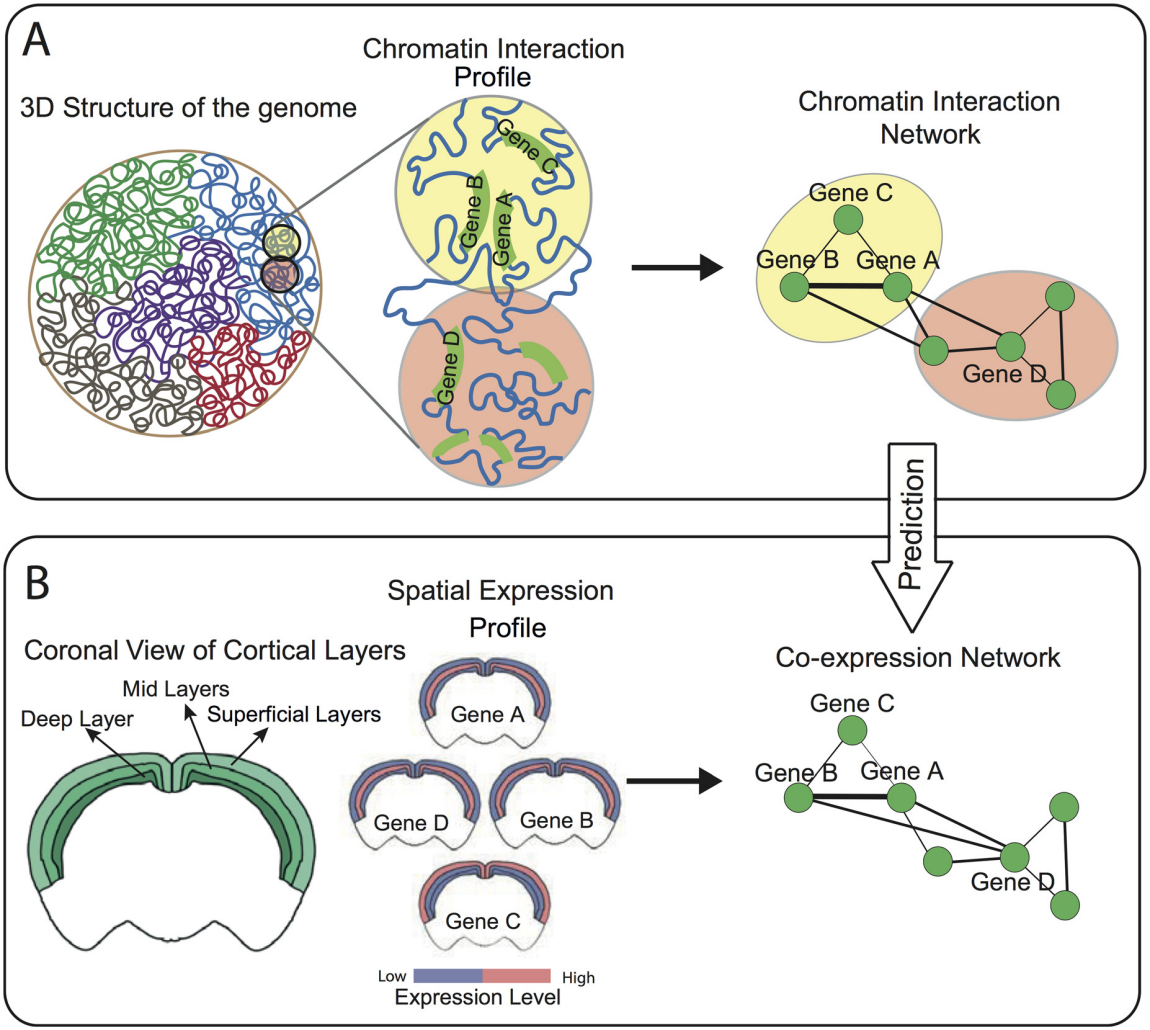
Association between chromatin interaction and co-expression of gene-pairs. (A) Co-regulated genes are co-localized in 3D structure of the genome through frequent chromatin interactions. Chromatin interactions can be at different levels from direct interaction between genes (interaction between Gene A and Gene B) to chromatin compartment interactions (interaction between Gene D and Gene B). Chromatin interactions between gene-pairs can be characterized by a network, termed Chromatin Interaction Network (CIN). (B) Co-expression between gene-pairs based on their spatial expression pattern across the mouse cortex. Gene A, Gene B and Gene D are expressed in the mid layers of the mouse cortex and are hence highly co-expressed. Gene C, on the other hand, is expressed in the superficial cortical layers and therefore is not co-expressed with the other three genes. The chromatin interaction profile of a gene-pair, encoded by the topological structure of the CIN, can be used to predict the co-expression status as captured by the co-expression network.

The CIN constructed in this study is based on Hi-C measurements from the mouse cortical cells [8]. In the brain, genes with a common expression pattern across the brain may have a common role in influencing the function of the brain region in which they are co-expressed [22]. In order to study spatial co-expression in the mouse brain, and mammals in general, it is necessary to map the expression at sufficient resolution to decode the high complexity [23]. The Allen Mouse Brain Atlas (ABA) [24], a genome-wide map of gene expression across the brain, provides sampled cellular-resolution *insitu* hybridization sections at a 25*μ*m interval across the entire brain. We use this high-resolution dataset to obtain spatial co-expression relationships between genes at the cellular level (Fig 1), i.e. two genes will be co-expressed if they are expressed in the same set of cells across the brain.

To test the hypothesis that co-expression in the cortex is encoded in the CIN, we employ a supervised learning procedure. More specifically, we aim to predict the spatial co-expression between gene-pairs based on a set of features that describe the topology of the connection between the two genes in the CIN. We show that the resolution at which the chromatin interactions are captured affects the prediction of co-expression from genomic organization. In particular, our results reveal that the accuracy of the prediction is increased when measures from different Hi-C resolutions are integrated. Finally, we clearly demonstrate the importance of using descriptions of the CIN topology at different scales, ranging from specific interactions between transcription start sites of genes (small-scale) through interactions between whole genes (medium-scale) and interaction between chromatin compartments (large-scale).

## Results

### Intra-chromosomal Hi-C data

We collected the intra-chromosomal Hi-C data from Shen et al. [8]. They obtained Hi-C measurements in the mouse cortex following the methods proposed in Lieberman-Aiden et al. [12]. About 20–30 million cortex cells from 8-week old male C57Bl/6 mice were used to generate Hi-C contact matrices [8]. The resulting Hi-C matrices contain pair-wise chromatin contact frequencies between pairs of 40*kb* genomic segments (i.e. bins). Experimental biases, such as GC content of trimmed ligation junctions and distance between restriction sites, were eliminated by an integrated probabilistic background model as described by Yaffe et al. [25]. Hi-C technology measures only steady-state chromosome conformations across a population of cells. So, the resulting genome-wide interactions are averaged across the cells and are not exactly the same in any given cell [8, 26]. Yet, the variability of chromatin interactions is mostly confined to local interactions, while long-range interactions are relatively well conserved and stable [27]. This demonstrates that different cell types share a common global architecture of their chromosomes which can be well described by the chromatin contact matrix.

Two regions that are close-by in the linear genome are expected to have higher chromatin interaction frequency, irrespective of the actual 3D organization of the genome (S1 Fig). To account for this, several studies [12, 28, 29] have defined normalized Hi-C contact matrices assuming that the Hi-C interactions are normally distributed [12, 28] or independent [29]. Alternatively, we used a non-parametric rank based normalization method [30] to describe the Hi-C score distributions for a certain distance, which we found to be more powerful for detecting variations across the genomic distance.

### Multi-resolution Hi-C data

Since we are interested in predicting co-expression patterns of genes, each bin-based Hi-C matrix is converted to a gene-based Hi-C matrix based on the Hi-C interaction between the corresponding bins in which the genes reside (see Methods). While assigning Hi-C interactions between genes, the bin size of the Hi-C data controls the genomic neighborhood considered around genes. In order to capture interactions between genes at variable linear genomic distances we varied the resolution of the Hi-C data matrices, before constructing gene-based matrices. This was achieved by considering different bin sizes between 40*kb* (high-resolution) and 1*Mb* (low-resolution). The lower resolution matrices were obtained by summing the contact frequencies of consecutive bins in the higher resolution matrices.

### Chromatin interaction network (CIN)

To determine the Hi-C interactions between each gene-pair we take the Hi-C interaction between the corresponding bins in which the genes reside. However, some genes might span multiple bins, depending on gene size and bin size. In this case, we determine the Hi-C interaction for a gene-pair (*x*, *y*) by one of two approaches. In the first approach, referred to as MAX-mapping, we define a link as the maximum Hi-C value among all possible interactions, i.e. ${\hat{h}}_{xy}=max_{i\in x,j\in y}({\hat{h}}_{ij})$. In the second approach, referred to as TSS-mapping, we define a link as the Hi-C score between the bin-pair which contains the transcription start sites (TSS) of the two genes, i.e. ${\hat{h}}_{xy}={\hat{h}}_{ij};$ where: *TSS*(*x*) ∈ *i* and *TSS*(*y*) ∈ *j*. We applied a threshold to convert the weighted gene-based Hi-C matrix to an un-weighted matrix by retaining only interactions that exceed the 90^*th*^-percentile of all Hi-C score across all chromosomes at the corresponding bin size.

We constructed one CIN per chromosome per resolution because the employed Hi-C data contains only intra-chromosomal interactions. For each CIN $H_{chr}^{R}=(G,I_{H})$, *G* represents the set of nodes corresponding to genes and *I*_*H*_ represents the set of links corresponding to Hi-C interactions between genes that exceed the 90^*th*^-percentile of all Hi-C scores across all chromosomes at a resolution *R*.

### CIN topology

There are several topological measures which capture graph structure for nodes and/or links in a network [17, 19]. In this work, we calculated five graph-topological measures of the chromatin interaction network: shortest path length, Jaccard index, degree (and closeness) centrality, betweenness centrality, and clustering coefficient (Table 1). Since our goal is to predict co-expression between gene-pairs, all features used by the classifier should be link-based. Therefore, we converted all the node-based topological measures (degree-closeness centrality, betweenness centrality and clustering coefficient) to link-based measures by taking the average and the difference between the values of the gene-based measure for each gene-pair. For example, for a gene-pair (*x*, *y*), the clustering coefficient of the link between *x* and *y* is described by $\left\{\left|(cc(x)-cc(y)\right|,\frac{1}{2}(cc(x)+cc(y))\right\}$. As a result, each link in the interaction network is represented by eight link-based topological features.

**Table 1:** Topological measures.

**Measure**	**Description**	**Scale-aware version**
Shortest Path	The minimum number of vertices connecting node *x* and *y*, *s*(*x*, *y*)	$s^{\beta }(x,y)=-\log(K_{x,y}^{\beta })$
Jaccard Index	The proportion of shared nodes between *x* and *y* relative to the total number of nodes connected to *x* or *y*, $J(x,y)=\frac{n(x)\cap n(y)}{n(x)\cup n(y)}$	$J^{\beta }(x,y)=\frac{\sum_{i}min(K_{x,i}^{\beta },K_{i,y}^{\beta })}{\sum_{i}max(K_{x,i}^{\beta },K_{i,y}^{\beta })}$
Degree & closeness Centrality	The degree centrality reflects the connectivity of a node in terms of the number of edges connected to it, *deg*(*x*) and closeness centrality reflects the farness of a node *x*, by summing the shortest path distances to all other nodes, $c(x)=\frac{1}{\sum_{i\backslash x}s(x,i)}$	$c^{\beta }(x)=1-K_{x,x}^{\beta }$
Betweenness Centrality	The number of shortest paths that pass through a node, $b(x)=\sum_{i,j\backslash x}\frac{q_{ij}(x)}{q_{ij}}$ where *q*_*ij*_ is the number of shortest paths between nodes *i* and *j*, and *q*_*ij*_(*x*) the number of those paths that pass through *x*	$b^{\beta }(z)=\frac{1}{N^{2}}\sum_{x,y}\left(s^{\beta }(x,y)-(s^{\beta }(x,z)+s^{\beta }(z,y))\right)$
Clustering Coefficient	The number of edges between its direct neighbors including itself, divided by the maximum number of possible edges, $cc(x)=\frac{2|e_{x}|}{deg(x)(deg(x)-1)}$	$cc^{\beta }(x)=\sum_{i\backslash x}K_{x,i}^{\beta }J^{\beta }(x,i)$

*N* is the set of all nodes in the network, and *n* is the number of nodes. (*x*, *y*) is a link between nodes *x* and *y*, (*x*, *y* ∈ *N*). *a*(*x*, *y*) is the connection status between *x* and *y*: *a*(*x*, *y*) = 1 when link (*x*, *y*) exists; *a*(*x*, *y*) = 0 otherwise. Scale-aware versions are based on diffusion kernel where *K* ^*β*^ = *e* ^*β*(*A* − *D*)^, *A* is the adjacency matrix and *D* is the degree matrix of the network. The diffusion level *β* determines the scale. *K* ^*β*^(*x*, *y*) is the diffusion strength between node *x* and *y*.

In addition to the standard topological measures, we used the scale-aware topological measures (STMs) described by Hulsman et al. [21] to capture the network characteristics across different scales. STMs are based on diffusion kernels [30], a network smoothing process in which the diffusion strength *β* parameter determines the scale at which the network is considered [31]. By varying the scale at which we consider the CIN, different types of interactions are taken into account. For example, specific interactions between transcription start sites of genes are more pronounced at the small-scale while interactions between chromatin compartments are more pronounced at the large-scale.

### Co-expression network

The Allen Mouse Brain Atlas (ABA) [24]; (http://mouse.brain-map.org/) provides a genome-wide cellular-resolution, *insitu* hybridization (ISH)-based, gene expression map of the 8–week old adult *C*57*BL*/6*J* male mouse brain. A spatial co-expression map was constructed based on the similarity of the spatial expression profiles of each pair of genes across the cortex (see Methods).

The employed Hi-C data contains only intra-chromosomal interactions. Therefore, one co-expression network was constructed per chromosome and is denoted by *E*_*chr*_ = (*G*, *I*_*E*_), where *G* indicates a set of nodes representing genes and *I*_*E*_ indicates set of links representing intra-chromosomal co-expressions between gene-pairs. The largest and smallest networks *E*_2_ and *E*_18_ (S2 Fig) consisted of 338 and 119 genes (i.e. nodes), respectively. To focus our predictions on reliable interactions, we included only strongly co-expressed genes and gene-pairs without strong co-expression (see Methods).

### Highly co-expressed genes are spatially co-localized

To examine whether gene-pairs with high spatial co-expression frequently interact in the 3D conformation of chromosomes, we defined two sets of gene-pairs: strongly co-expressed genes and gene-pairs without strong co-expression (see Methods). We used a Wilcoxon rank-sum test to determine if strongly co-expressed gene-pairs have stronger Hi-C interactions, and hence are closer to each other in the 3D conformation of the chromosome, compared to gene-pairs without strong co-expression.

**Fig 2:**
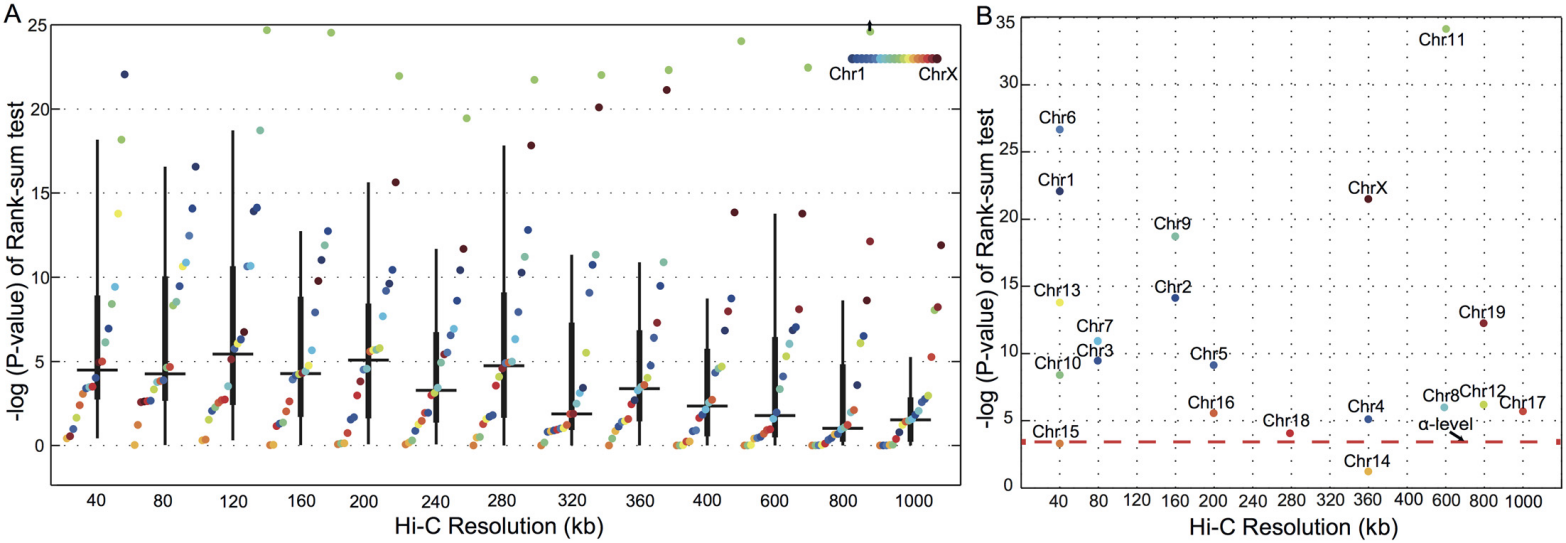
Co-expressed genes are co-localized in 3D structure of the genome. (A) Assessment of the enrichment of Hi-C interactions between strongly co-expressed gene-pairs compared to gene-pairs with no co-expression across different Hi-C resolutions. The y-axis indicates −*log*_10_(*p* − *value*) of the one-tailed Wilcoxon rank-sum test used for the enrichment analysis. Hi-C interactions were mapped to genes using the MAX-mapping method. (B) Overview of the Hi-C resolution at which Hi-C interactions are most significantly associated with co-expressed gene-pairs for each chromosome. In each box, the horizontal line represents the median. The thick vertical line represents the interval of *q*_1_ = 25^*th*^ and *q*_3_ = 75^*th*^ percentiles. The thin vertical line represents the interval of *q*_3_ + 1.5(*q*_3_ − *q*_1_) and *q*_1_ − 1.5(*q*_3_ − *q*_1_).

Fig 2A (and S3 Fig) shows that co-expressed genes are significantly co-localized in the nucleus in most of the chromosomes and most CIN-resolutions (Wilcoxon rank-sum test; *p*-value < 0.0002, Bonferroni corrected for 260 tests: 20 chromosomes × 13 resolutions). Strikingly, we observe that the resolution for which the strongest co-localization is attained is different for different chromosomes (Fig 2B). This observation underscores the importance of a multi-resolution approach to characterize chromatin interactions which apparently can occur between loci in the direct vicinity of genes as well as between broader regions (domains) in which these genes reside.

### Chromatin interaction profiles as co-expression predictors

To determine whether strong co-expression can be predicted from chromatin interactions, we calculated the correlation between the Hi-C matrix and the co-expression matrix for each chromosome at different resolutions. S4 Fig shows that the correlation is very low across different chromosomes and Hi-C resolutions (−0.4 to +0.1). Additionally, training a classifier on the presence or absence of links in the CIN results in a poor classification performance (0.55 median AUC across chromosomes at 40*kb* resolution). S5 Fig shows that only 2% (average across all chromosomes) of all gene-pairs are co-expressed and connected in the CIN of each chromosome. This observation further highlights the importance of indirect chromatin interactions in explaining co-expression. Taken together, these results indicate that chromatin interaction and co-expression do not have an injective (one-to-one) relation. The relation between chromatin interaction and co-expression would be better described by a more comprehensive characterization of long-range interactions, i.e. indirect interactions.

**Fig 3:**
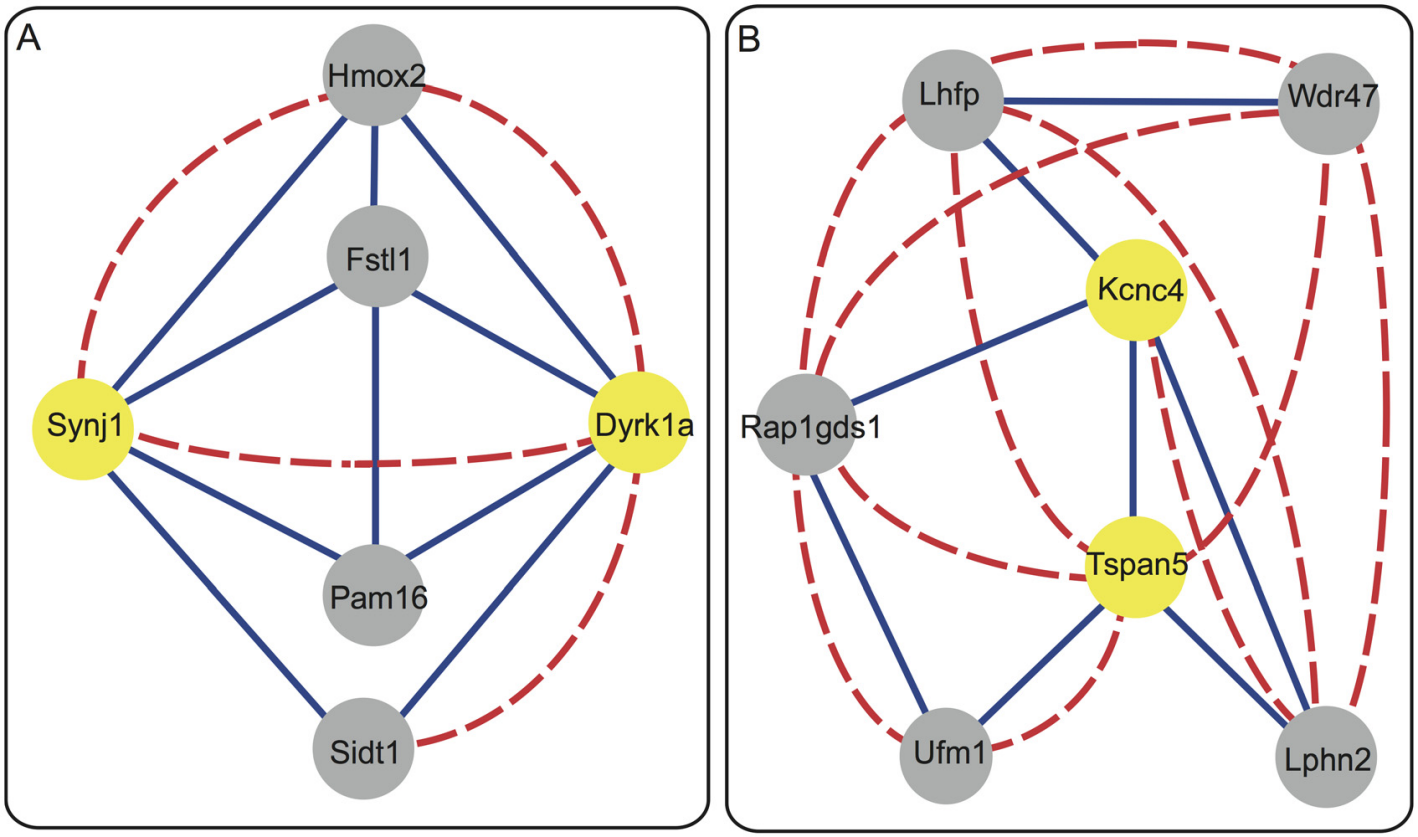
Chromatin interactions of gene-pairs in the CIN at 200*kb* resolution. (A) *Synj*1-*Dykr*1*a* (yellow nodes) in Chromosome 16 are co-expressed (dashed red link) but their corresponding genomic loci do not interact frequently (no blue link). Both genes have strong chromatin interactions with 4 other genes (grey nodes) resulting a high Jaccard index between them. (B) *Kcnc*4-*Tapan*5 (yellow nodes) in Chromosome 3 directly interact (solid blue line) but they are not strongly co-expressed (no dashed red line). This direct chromatin interaction explains the strong co-expression between other gene-pairs in their neighbourhood, such as *Wdr*47-*Tapan*5 and *Wdr*47-*Rap*1*gds*1, which are not directly connected in the CIN themselves (no solid blue line). The betweenness centrality measure of the link between *Kcnc*4-*Tapan*5 can describe the strong co-expression between their neighbouring genes. Chromatin interaction and co-expression are shown by solid blue and dashed red links, respectively.

A compelling example is given in Fig 3A. In Chromosome 16, *Synj*1 and *Dyrk*1*a* genes are co-expressed (dashed red line) while their corresponding genomic loci do not frequently interact, i.e. there is no link (solid blue line) between them in the CIN at 200*kb* resolution. A classifier only taking direct chromatin interactions into account will mistakenly predict that the two genes are not co-expressed. However, both *Synj*1 and *Dyrk*1*a* genes have strong chromatin interactions with *Pam*16, *Fstl*1, *Hmox*2, *Sidt*1 and their strong co-expression can be correctly predicted if these indirect interactions are considered. For this particular example, the indirect interactions between the two genes can be characterized by the Jaccard index which captures to what extent the two genes have common direct neighbors.

Another example is the interaction between *Kcnc*4 and *Tspan*5 in Chromosome 3 (Fig 3B). *Kcnc*4 and *Tspan*5 directly interact in the 200*kb*-CIN (solid blue line) but they are not strongly co-expressed (no dashed red line). Nevertheless, this direct chromatin interaction may explain the strong co-expression between gene-pairs in the CIN neighborhood that lack a direct chromatin interaction themselves. For example, *Wdr*47 and *Lphn*2 are co-expressed although they are not directly connected in the CIN (no solid blue line) but their co-expression can be explained by the chromatin interaction path through the *Kcnc*4, *Tspan*5 and *Tspan*5 genes. Similarly, the co-expression of *Wdr*47 and *Rap*1*gds*1 can be explained by the chromatin interaction path through *Kcnc*4 and *Tspan*5. For this example, the importance of the Hi-C link between *Kcnc*4 and *Tspan*5 to describe strong co-expression between their neighboring genes in the CIN can be captured using the betweenness centrality of both genes. Both examples illustrate that strong co-expression between gene-pairs can be better explained by their chromatin interaction profile, defined as the path connecting two genes in the context of the CIN.

### Topological descriptions of multi-resolution interaction networks increase the prediction performance

**Fig 4:**
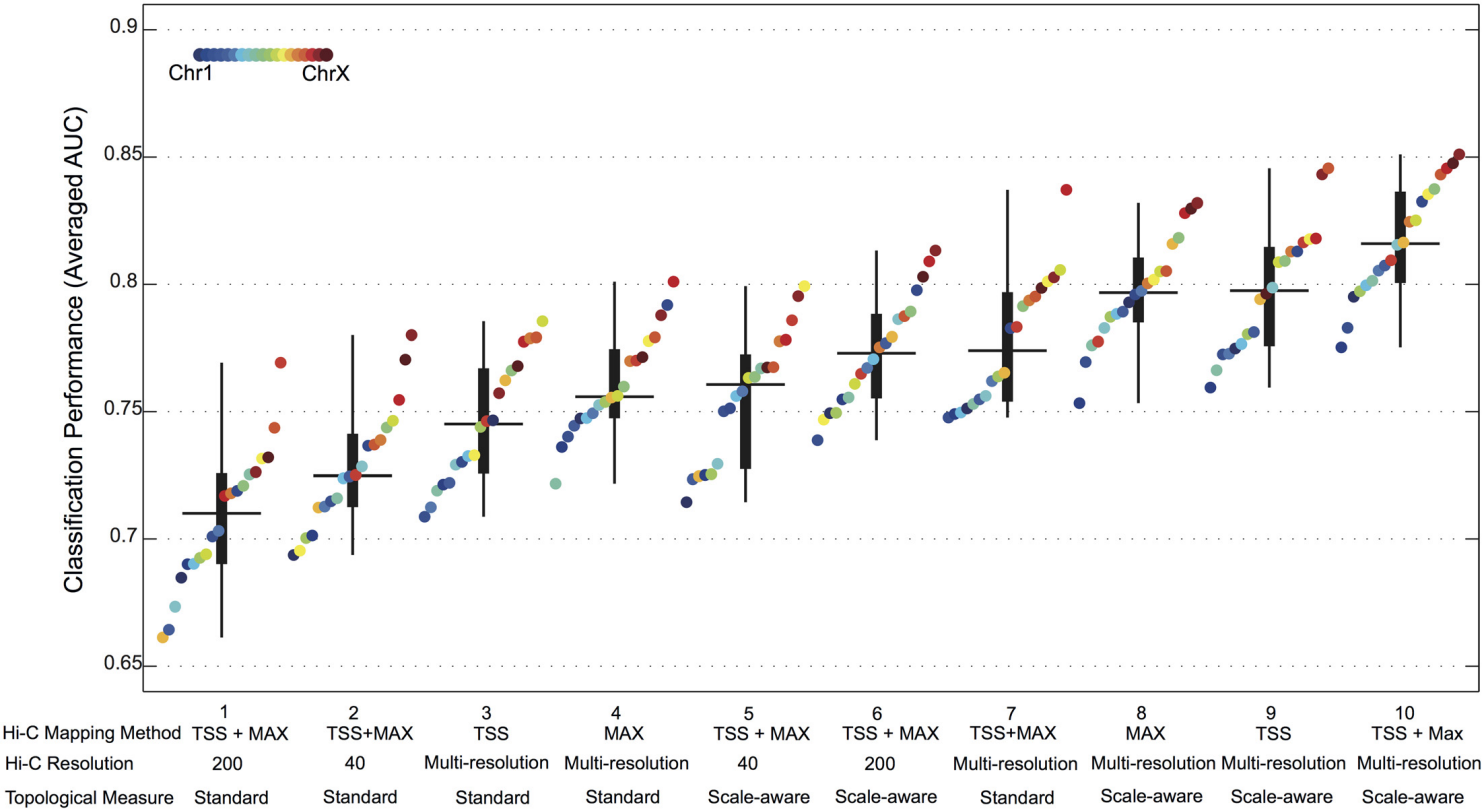
Classification performance for the co-expression prediction based on intra-chromosomal chromatin interaction networks. Each box encompasses the classifier performance in terms of AUC for all mouse chromosomes. Boxes are sorted based on their medians. The method that was used for computing the input feature set is given under each box. TSS or MAX refers to the mapping method for assigning Hi-C interaction between gene-pairs when the CIN is built. TSS + MAX refers to concatenated feature set of topological measures of CINs built using both TSS- and MAX-mapping methods. Multi-resolution refers to concatenated feature set of topological measures obtained from CINs at Hi-C resolutions of 40, 80, 120, 160, and 200*kb*. In each box, the horizontal line represents the median. The thick vertical line represents the interval of *q*_1_ = 25^*th*^ and *q*_3_ = 75^*th*^ percentiles. The thin vertical line represents the interval of *q*_3_ + 1.5(*q*_3_ − *q*_1_) and *q*_1_ − 1.5(*q*_3_ − *q*_1_). All the values shown in the figure are also available in S1 Table

For each CIN of a certain resolution, we calculated the standard graph-topological measures and trained a random neural network (RNN) classifier using the resulting topological features (see Methods). The classification results are summarized in Fig 4 (Box 1–4 and 7). The figure shows that an increased—yet moderate—classification performance is obtained when standard topological measures of the CIN at a single resolution (median AUC of 0.72 for 200*kb* and 0.73 for 40*kb*, Fig 4 (Box 1, 2)) are used as features (compared to 0.55 AUC when using only direct interactions).

To evaluate the effect of Hi-C resolution on co-expression prediction, we applied the RNN classifier to a concatenated set of standard topological measures obtained from CINs at different Hi-C resolutions (40, 80, 120, 160, and 200*kb*), i.e. the topological descriptions of each resolution are concatenated in one feature representation. At a high Hi-C resolution (40*kb*) we mainly focus on chromatin interactions between pairs of genes. On the other hand, at a low Hi-C resolution (200*kb*) we consider interactions between larger genomic domains. Our multi-resolution approach increased the power of the interaction data to predict co-expression (Fig 4, Box 3, 4, 7), supporting our earlier observation that gene regulation occurs at different regional scales of chromatin interaction, such as the gene-level or the level of broad regions. So far, the best prediction performance is obtained by concatenating standard topological measures of CINs built using both TSS- and MAX-mapping methods (0.77 median AUC, Fig 4, Box 7).

### STMs improve the prediction performance

To examine the effect of indirect chromatin interactions on the prediction of co-expression, we described the CIN topology at multiple topological scales using STMs (see Methods). We calculated STMs of the CIN at each Hi-C resolution separately and then concatenated all STMs, resulting in 800 features; 8 STMs at 10 scales applied to 10 CINs; 5 different resolutions and two mapping methods (see Methods for more details). We then followed the same procedure as before and trained a RNN classifier on this combined feature set.

Fig 4 (Box 5–6 and 8–10) summarizes the results obtained when using STMs rather than the standard topological measures. The performance obtained using STMs calculated at a single resolution CIN (Fig 4, Box 5, 6) is comparable to the performance obtained by concatenating standard topological measures from multi-resolution networks (Fig 4, Box 7). However, by combining features from STMs applied to multi-resolution CINs, the power to predict co-expression improves significantly (Wilcoxon rank-sum test; *p*-value < 0.00001) (0.82 AUC, Fig 4, Box 10). The best performances are obtained for Chromosome 16 (0.86 AUC) and Chromosome X (0.85 AUC). The observed performance improvement demonstrates that it is important to use a scale-aware topological description of the CIN to capture the complex 3D organizational features of the genome that determine gene co-expression.

In order to analyze the effect of considering only strongly co-expressed genes on the classification performance, we assessed the performance when all co-expression links are included. In this analysis, a gene-pair is labeled co-expressed (i.e. positive class) or not co-expressed (i.e. negative class) if their correlation is above or below the median (i.e. 50^*th*^-percentile) of all correlations across all chromosomes, respectively. The resulting AUCs across all chromosomes show that STMs performs better than standard measures to distinguish between co-expressed and no co-expressed gene-pairs (S6 Fig). As expected, the classification performance is lower with respect to the case where we excluded weakly co-expressed gene-pairs (i.e. gene-pairs that have a co-expression that is in between the 50^*th*^ and 90^*th*^-percentile of all correlations across all chromosomes) (Fig 4). Most likely this is caused by a noisy class assignment for weakly correlated gene-pairs which confuses the classifier during training.

We also performed the classification procedure by including Hi-C scores above the median of all Hi-C scores across all chromosomes. The resulting AUCs across all chromosomes show that STMs perform better than standard measures to distinguish between co-expressed and non-co-expressed gene-pairs (S6 Fig). The classification performance is, however, less than the AUC when we defined strong Hi-C interactions as Hi-C scores above the 90^*th*^-percentile of all Hi-C scores across all chromosomes (Fig 4).

To compare the rank-based normalization method [32] with the average-based method proposed by Lieberman et al. [12], we trained the classifier on the standard and scale-aware topological measures of the CIN that was built using the average-based normalized Hi-C matrices. The performance of these classifiers is lower than when constructing the CIN on using the rank-based normalized Hi-C data (S7 Fig), underscoring the usefulness of the rank-based normalization for predicting co-expression from chromatin interaction data. Nevertheless, STMs perform better than standard measures for both normalization methods, indicating that the classifier is not biased towards the normalization method.

To investigate the effect of chromatin interactions between non-genic and genic regions on the co-expression prediction we built a bin-based CIN (instead of a gene-based CIN). In the bin-based CIN, nodes represent non-overlapping bins with size of 200*kb* and links represent Hi-C interactions between bins that exceed the 90^*th*^-percentile of all Hi-C scores across all chromosomes at a 200*kb* resolution. We calculated standard and scale-aware topological measures (8 measures) for all links in the bin-based CIN. The classifier was trained on topological measures of the portion of links that connect two gene-loci. In this strategy, the interaction profile between two gene-loci is characterized by chromatin interactions of all genomic regions across the scales. The resulting AUCs across all chromosomes show that STMs performs better than standard measures to distinguish between co-expressed and non-co-expressed gene-pairs (S8 Fig). It is interesting to observe that the classification performance is approximately similar to that obtained when gene-based CINs were used. This suggests that the STMs can capture all the necessary information from the genic Hi-C links.

### CIN topology differs per chromosome

To investigate the variation in topological properties of the CIN of different chromosomes, we performed a leave-one-chromosome-out experiment. If the CINs of all 20 mouse chromosomes share the same topological properties, then it would be possible for a classifier trained on all but one chromosome to accurately predict the co-expression labels of the left-out chromosome. To test this hypothesis, we trained the RNN classifier on the STMs (800 features) extracted from 19 chromosomes and then tested the performance on the left-out chromosome. We repeated the procedure 20 times and each time, a different chromosome was left out of training and used for testing. The maximum AUC obtained was 0.54, which indicates that the CIN of each chromosome has a unique topology, to which the high-scale STM feature values are sensitive.

The variation in topological properties of CINs across chromosomes is also observed when we trained an RNN classifier on individual topological measures. The classification performance using individual standard measures (S9 Fig) and individual STMs (S10 Fig) is highly variable across chromosomes, which explains the poor performance obtained in the leave-one-chromosome-out experiment. For instance, the clustering coefficient STM is a good descriptor of the CIN of Chromosome 3 at medium-resolution and low-scale, while it is a good descriptor of the CIN of Chromosome 10 at high-resolution across the scales (S10 Fig).

### Topological signatures of CINs

To analyze the topological properties that are most predictive we trained the classifier on individual topological measures. The classification performance using individual standard measures (S9 Fig) and individual STMs (S10 Fig) shows that none of the topological measures has dominant power to predict co-expression. Therefore, the classifier requires more than a single topological descriptor to describe chromatin interaction profile between two gene-loci. In order to determine the set of STMs that characterizes the CIN of each chromosome the best, we performed forward feature selection in combination with the RNN classifier. To facilitate this computationally, we reduced the number of nodes in the hidden layer to 100 and applied 5-fold cross validation. To ease interpretation, we used the STMs derived from multi-resolution CINs using the MAX-mapping method only (400 STMs, 8 measures × 5 resolutions × 10 scales). S11 Fig shows that the classification performance achieved using feature selection (0.8 AUC) is higher than the performance achieved using all features (0.72 AUC). For most chromosomes, the top 5 selected features in all 5 folds are clustering coefficient (at small-scale, *β* < 0.5), closeness centrality (at medium-scale (0.5 < *β* < 3) and Jaccard index (at large-scale, *β* > 3) STMs (S2 Table).

The clustering coefficient measures to what extent a gene is embedded in a well-connected component of the CIN. Selecting the small-scale clustering coefficient implies that co-expressed genes are usually embedded in a locally well-connected component in the CIN (e.g. chromatin compartment). The Jaccard index determines the fraction of common interacting genes between gene-pairs in the CIN. At a large scale it takes more indirect neighboring nodes (e.g. genes located in different chromatin compartments) into account. The closeness centrality reflects the farness of a gene by summing the shortest path distances to all other genes and at a medium scale it thus takes somewhat longer paths into account. Both Jaccard index and closeness centrality explain that common indirect interacting genes (e.g. interaction between chromatin compartments) are important to describe the co-expression pattern of a pair of genes.

Additionally, we observed that all scale-levels (small, medium and large) were selected reflecting the importance of characterizing CINs at different scales. The selection of various scale-levels could be explained by the hierarchical structure of the chromatin folding in the cell nucleus ranging from looping between the promoter regions of genes to larger chromatin compartments [11, 15]. This is corroborated in the work by Sandhu et al. [15] who have shown that genomic regions are organized into a hierarchical chromatin interaction network.

### STMs effectively characterize the CIN of Chromosome 16 to predict co-expression

We analyzed the top selected STMs of the 200*kb*-CIN of Chromosome 16, for which the highest prediction performance is achieved, to gain insight into the topological measures and scales that best describe the network. The best classification performance (AUC = 0.84) is obtained using 206 of the 400 STMs (S2 Table) which are selected by forward feature selection. We mapped these 206 features to a 2D space using t-Distributed Stochastic Neighbor Embedding (t-SNE) [33, 34] (see Methods).

**Fig 5:**
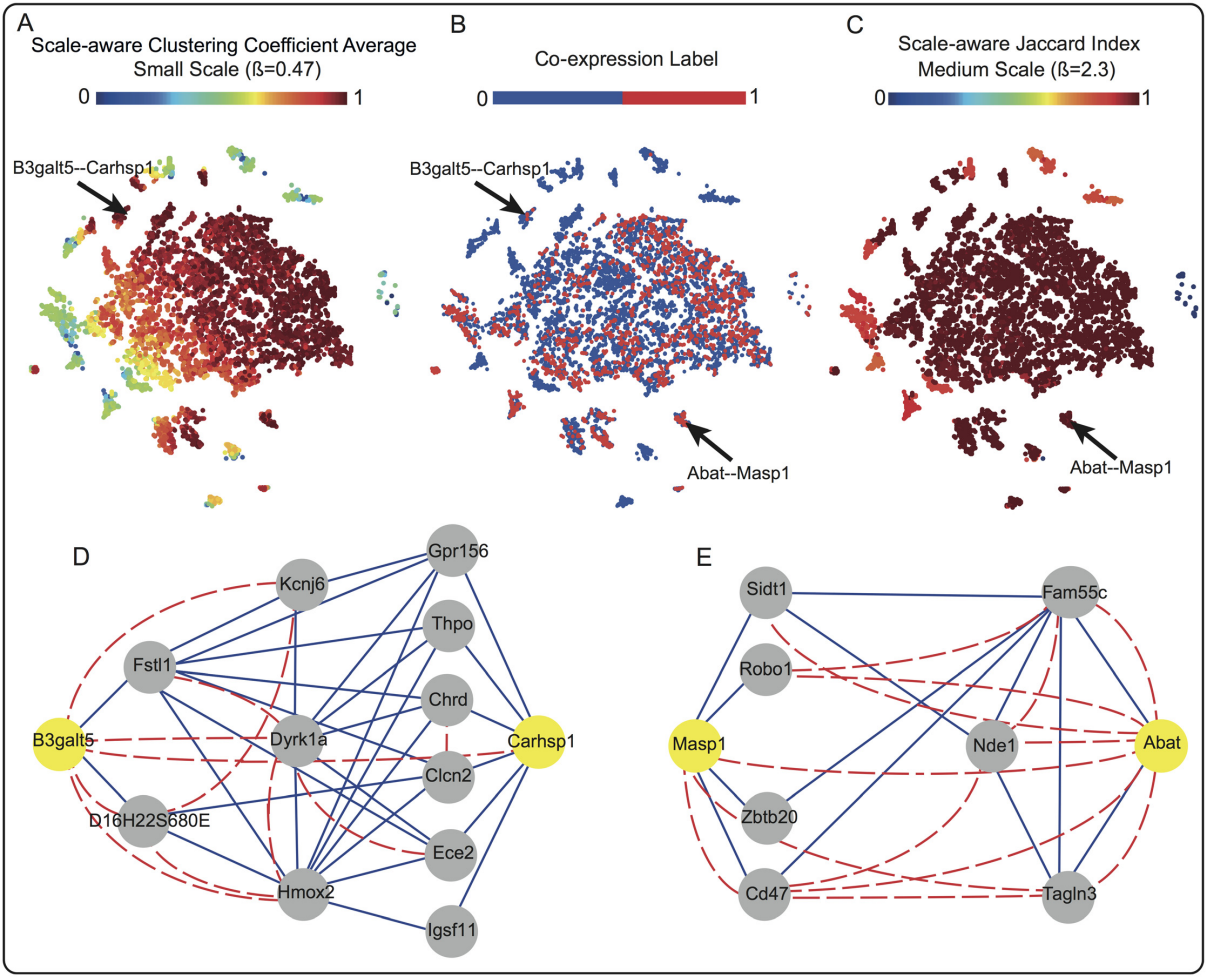
Topological signature of Chromosome 16 CIN. t-SNE maps of the 200*kb*-CIN of Chromosome 16. Each point in the map corresponds to a link between a gene-pair which is colored according to (A) Clustering coefficient at small-scale, (B) Co-expression label, and (C) Jaccard index at the medium-scale. (D) and (E) show sub-networks of the CIN surrounding selected gene-pairs (indicated in the 2D maps): (D) *B*3*galt*5-*Carhsp*1 (yellow nodes) with the high clustering coefficient average; and (E) *Masp*1 and *Abat* (yellow nodes) with the high Jaccard index. Chromatin interaction and co-expression are shown by solid blue and dashed red links, respectively.

The 2D map of all gene pairs in Chromosome 16 (Fig 5) shows that there are few distinct clusters of co-expressed and not co-expressed gene-pairs, i.e. clustering of red and blue dots in Fig 5B respectively. However, it is difficult to discriminate between the majority of gene-pairs (big cluster in the middle of Fig 5B), further supporting our observation of complex organization of chromatin interactions. Coloring the t-SNE with two of the top selected features, the clustering coefficient at small-scale (Fig 5A) and the Jaccard index at the medium-scale (Fig 5C), shows that gene pairs are characterized by different values of those two features, indicating their importance for the classification performance.

Since the clustering coefficient at small-scale is one of the top selected features for Chromosome 16, we used the t-SNE map to select a co-expressed gene-pair with a high clustering coefficient at a small-scale (Fig 5A). We constructed a sub-network of the selected gene-pair by retrieving all the Hi-C and co-expression interactions surrounding the gene-pair (Fig 5D). *B*3*galt*5 and *Carhsp*1 are co-expressed (dashed red link in Fig 5D and red dot in Fig 5B) although there is no direct Hi-C interaction between them (no blue link). However, it is possible to predict their co-expression because they are both part of a very well connected cluster, which is captured by a high average clustering coefficient at small scale.

Similarly, we select a co-expressed gene-pair with a high Jaccard index at the medium-scale, another top selected STM of the CIN of Chromosome 16 (Fig 5C). The sub-network including the selected gene-pair *Masp*1 and *Abat* (Fig 5E), shows that they are co-expressed although no direct Hi-C interaction exists between them (no blue link in Fig 5E). The two genes also do not share many direct neighbors. At a medium scale, however, the Jaccard STM takes indirect neighbors into account, resulting in a high Jaccard index based on the Hi-C links between the neighbors of *Masp*1 and *Abat*.

## Discussion

We proposed a network-based approach to better understand the 3D structure of the genome based on scale-aware topological measures of the chromatin interaction network. Previous studies have shown a strong correlation between co-expression and chromatin interaction, for example in model organisms (*e*.*g*. yeast) [3] or cell lines (human gm06990 and K562 cells) [2]. Our results demonstrate that the co-expression relationship between a pair of genes in the mouse cortex could be accurately predicted from their chromatin interaction profile, extending previous observations in [2, 3]. Furthermore, the predictive power of our model depends greatly on the resolution at which the interactions are observed as well as the scale at which the topological properties on the interaction network are calculated. By integrating scale-aware topological measures at multiple Hi-C resolutions, we were able to predict spatial co-expression between gene-pairs with an AUC performance of 0.82. To our knowledge, this is the first attempt to predict co-expression based on genome-wide chromatin interactions.

The results also showed a general trend of the prediction performance (Fig 4) suggesting that STMs across multiple Hi-C resolutions are necessary to accurately capture the 3D structural features in the genome that determine spatial co-expression between genes in the mouse cortex. While the multi-resolution approach captures direct chromatin interactions between genes at variable linear genomic distances, standard topological measures extracted from a single-resolution CIN fail to represent the complex 3D structure of genome. By using STMs [21] to describe each single-resolution CIN, we were able to capture both direct and indirect interactions between genes, and hence correctly predict their co-expression status.

The 2D t-SNE maps of the CINs using 80 standard topological measures (S12 Fig) and 800 STMs (S13 Fig) reveal a complex organization of chromatin interactions, indicating that the discrimination between co-expression labels (blue and red points in S12 Fig and S13 Fig) is a difficult task. These observations may also explain the poor classification performance obtained using a simple classifier such as nearest mean (NM). The RNN classifier, however, is able to capture the complex chromatin interaction profile of a gene-pair and their co-expression status.

Comparing the t-SNE map of standard topological measures and STMs of Chromosome 16’s CIN shows that STMs are indeed more powerful in discriminating co-expression labels (S14 Fig). For example, the t-SNE map of standard topological measures shows that most of the interactions in the CIN of Chromosome 16 are characterized by a low Jaccard index value and consequently, the contribution of the Jaccard index to the classification performance is very low (S14 Fig). The scale-aware Jaccard index, however, captures indirect neighbors between a gene-pair which improves the classification performance.

Furthermore, we showed that each STM characterizes the CIN differently across scales and resolutions. For instance, the t-SNE map of STMs shows that the chromatin interaction profiles between gene-pairs in a well-connected component, indicated by a high clustering coefficient, are better captured at low resolution, whereas other well-connected components are better characterized at the high-resolution (different color pattern in S14 Fig). Additionally, some interactions are well discriminated using the clustering coefficient (a node-based STM) while other interactions are better discriminated using the Jaccard index (a link-based STM) (S14 Fig). This highlights the importance of both link- and node-based STMs in characterizing the topology of connectivity and neighborhood, respectively, of gene-pairs in the CIN to predict co-expression.

Our observations are in line with the two complementary models of how regulatory elements, such as enhancers and insulators, act to regulate the expression of distant genes [35]. The looping model assumes that loops along the genome are formed to bring distal regulatory sequences in direct contact with the promoters of target genes. Alternatively, genes undergoing transcription might co-localize in the nucleus in transcription factories, and enhancers facilitate the movement of genes into or out of these factories. Our finding that a multi-resolution scale-aware encoding of the CIN topology better predicts co-expression indeed shows that chromatin interactions occur at different levels, ranging from direct interactions between the transcription start sites of genes (small-scale) through interactions between genes (medium-scale) up to interaction between chromatin compartments (large-scale).

The topology of different chromosomes might be radically different, due to both chromosome length and different fractions of chromatin types. High-scale STM values are in particular sensitive to such a change in topology, and are likely to be one of the causes for the differences in performance. Indeed, a classifier, such as the one proposed here, might also be used to characterize chromatin conformation.

In the current study, we used only intra-chromosomal interactions. Nevertheless, our proposed methods could principally be applied to inter-chromosomal interactions given that the data is normalized properly across chromosomes [25, 36]. Furthermore, the method is not tissue- or organism-specific and can be generalized to predict any functional relationships (not only co-expression) between genomic loci (bins or genes) based on the characterization of the CIN.

The brain is a very complex structure with large variability in gene expression patterns across different regions. Using the high-resolution maps of the ABA, this variability could be used to identify distinct groups of genes with a similar expression pattern indicating their functional similarity [37, 38]. For example, several studies analyzed the relationship between spatial-co-expression and connectivity in the mouse brain [39–42]. Menashe et al. [23] used a spatial co-expression network of the mouse brain to identify common neuro-functional properties of autism-related genes. We expect that within the brain, and especially the cortex, many genes vary and that their biologically meaningful spatial correlation patterns are reflected by long-range chromatin interactions.

With the recent association of dozens of mutations in chromatin regulators to neuropsychiatric disorders [43], our method provides a promising approach to investigate the effect of those regulators on the cortical regulatory network. A good characterization of interactions in the CIN and their relationship to co-expression can add to our understanding of the genetic etiology of these diseases.

## Materials and Methods

### Rank-based normalization of Hi-C contact matrices

In order to eliminate genomic distance bias in a Hi-C matrix, each Hi-C contact value is replaced by its relative rank compared to Hi-C contacts between bins with a similar genomic distance, measured in base-pairs [32]. The normalized Hi-C score ${\hat{c}}_{ij}$ is defined as the rank of *c*_*ij*_ in the vector *C* ^*d*^, where *c*_*ij*_ is the Hi-C contact between bin *i* and *j* with genomic distance of *d* base pairs (bp). The vector *C* ^*d*^ is the *m*th super-diagonal of the Hi-C contact matrix with $m=\frac{d}{binsize}$ which contains Hi-C scores between all bin pairs that have the same genomic distance *d*. Ranks are adjusted for ties by using the average rank whenever values in *C* ^*d*^ are tied.

Note that by increasing the genomic distance, the length of *C* ^*d*^ decreases. Therefore, *C* ^*d*^ *s* are extended to have an equal length *L*. The extension is done by adding elements from *n* neighboring super-diagonals around *m*th super-diagonal to reach the constant length *L*. As we move further from the main diagonal, the number of elements on the *m*th super-diagonal becomes very small. Therefore, a substantial number of elements from neighboring super-diagonals are included. This is acceptable since the distributions of *C* ^*d*^ are more similar for large *d*, and can thus be pooled. We set *L* equal for all chromosomes to determine a genome-wide threshold of strong Hi-C scores between gene-loci. So, the normalized Hi-C scores (i.e. ranks) are set to be in the same range across all chromosomes. We set *L* to be equal to twice the number of bins on Chromosome 1, the largest chromosome in the mouse genome.

### Scale-aware topological measures

STMs were acquired by calculating the five topological measures described in Table 1 on a diffused network, across a range of scales (*β*). We empirically choose 10 values for beta in range of [0, 10] according to:

$$\begin{array}{c} \beta =\frac{2^{6b}-1}{2^{6}-1}\times \left(10-0.0001\right)+0.0001 \end{array}$$

with *b* = 0.0, …, 1.0 in 10 steps resulting *β*:[0.0001, 0.09, 0.24, 0.47, 0.8, 1.4, 2.3, 3.8, 6.2, 10]. As a result, for the scale-aware classification, 80 features (8 measures × 10 scales) were extracted from the chromatin interaction network.

### Spatially-mapped gene expression data

We downloaded all the expression energy volumes of the 4, 345 genes with coronal experiments from (http://mouse.brain-map.org/) [24], using the ABA Application Programming Interface (API). Expression energy is a measurement combining the expression level, defined as the integrated amount of signal within each voxel, and the expression density, defined as the amount of expressing cells within each voxel. We selected all voxels belonging to the cortex, defined as *Isocortex* in the ABA, and all the RefSeq genes, resulting in an expression matrix of 15, 410 rows (voxels) and 4, 230 columns (genes). We used SpearmanÍs Rank correlation as a measure of similarity between the spatial expression profiles of each pair of genes, resulting in a 4, 230 × 4, 230 spatial co-expression matrix. Gene entries from the spatial co-expression matrix were mapped to their genomic locations to determine the Hi-C contact frequency between gene-pairs based on the mouse reference genome (mm9: NCBI m37, *GCA*_000001635_.18).

We considered a gene-pair to be strongly co-expressed (i.e. positive label) if their correlation exceeds the 90^*th*^-percentile of all correlations across all chromosomes. Conversely, gene-pairs are considered to be without strong co-expression (i.e. negative label) when their correlation falls below the median of all correlations across all chromosomes.

### Supervised learning procedure

We used a random neural network (RNN) classifier from the PRTools toolbox [44] (Matlab 2012b) to predict the co-expression label of gene pairs using the topological measures of link connecting them in the CIN as features. RNN is a feed-forward neural network with one hidden layer. We set the number of hidden nodes to 800, the maximum number of input features (8 STMs at 10 scales applied to 10 CINs; 5 different resolutions and two mapping methods).

The performance of the classifier was determined using 10-fold cross validation and reported in terms of the area under the ROC (receiver operating characteristic) curve (AUC). The ROC curve represents the true positive rate (sensitivity) as a function of the false positive rate (1—specificity) for different discrimination thresholds of the classifier (S15 Fig). An AUC of 1 represents a perfect classification and 0.5 represent a random classification.

### t-SNE map

t-Distributed Stochastic Neighbor Embedding (t-SNE) [33, 34] was used to map the links of each chromosome’s CIN to a 2D space by reducing the dimensionality of the *N* × *M* data, where *N* is the number of gene-pairs in each chromosome and *M* is the number of topological features. In the resulting map, each Hi-C link is represented by a point in the 2D space where the distance between points reflect the similarity between their corresponding topological profiles. We applied t-SNE with perplexity of 30 and initial dimensionality reduction using 50 principal components.

## Supporting Information

S1 Table Classification performance for the co-expression prediction based on intra-chromosomal chromatin interaction networks. This table is illustrated in Fig 4.
(XLSX)

S2 Table Top selected feature using forward feature selection. Each sheet in the excel file includes top selected features of one mouse chromosome. The resulting AUC after the feature selection is also reported on the sheet name. For each chromosome, three sets of features are given: top 5, top 10 and all selected features that result in the best performance. Features are included in each of the 3 sets if they are selected in at least one of the 5 folds. A feature is indicated by the type of topological measure, the scale and the resolution of the CIN at which it is calculated.
(XLSX)

S1 Fig Rank-normalized Hi-C contact matrix. (A) Hi-C contact matrix of Chromosome 16. (B) Rank-normalized Hi-C contact matrix. The genomic distance bias in the Hi-C contact matrix is eliminated by using a rank-based normalization (described in the main text).
(TIF)

S2 Fig Number of genes included in the CIN of each chromosome. (TIF)

S3 Fig Co-expressed genes are co-localized in 3D structure of the genome. (A) Assessment of the enrichment of Hi-C interactions between strongly co-expressed gene-pairs compared to gene-pairs with no co-expression across different Hi-C resolutions. The y-axis indicates −*log*_10_(*p* − *value*) of the one-tailed Wilcoxon rank-sum test used for the enrichment analysis. Hi-C interactions were mapped to genes using the TSS-mapping method. (B) Overview of the Hi-C resolution at which Hi-C interactions are most significantly associated with co-expressed gene-pairs for each chromosome. In each box, the horizontal line represents the median. The thick vertical line represents the interval of *q*_1_ = 25^*th*^ and *q*_3_ = 75^*th*^ percentiles. The thin vertical line represents the interval of *q*_3_ + 1.5(*q*_3_ − *q*_1_) and *q*_1_ − 1.5(*q*_3_ − *q*_1_).
(TIF)

S4 Fig Correlation between Hi-C and co-expression matrices. The Pearson’s correlation coefficient between the Hi-C matrix and co-expression matrix of all gene-pairs in each chromosome. Each box represents the correlations for all mouse chromosomes at a specific Hi-C resolution. Hi-C interactions between genes were determined using (A) the MAX-mapping method and (B) the TSS-mapping method. In each box, the horizontal line represents the median. The thick vertical line represents the interval of *q*_1_ = 25^*th*^ and *q*_3_ = 75^*th*^ percentiles. The thin vertical line represents the interval of *q*_3_ + 1.5(*q*_3_ − *q*_1_) and *q*_1_ − 1.5(*q*_3_ − *q*_1_).
(TIF)

S5 Fig Percentage of gene-pairs that (not)co-expressed with(without) a direct chromatin interaction in each chromosome. Percentage of interacting genes that co-express, the percentage of interacting genes that do not co-express, and the percentage of non-interacting genes that co-express per chromosome in CINs at A) 200*kb*, B) 40*kb* resolution. The percentage of gene-pairs with either a Hi-C link or co-expressed is about 22% (average across all chromosomes). Additionally, we also observed the percentage of co-expressed gene-pairs with a Hi-C link is very low per chromosome (2% average across all chromosomes).
(TIF)

S6 Fig Classification performance using different thresholds of co-expression and Hi-C. A) Classification performance using *all* co-expression links. Classification performance in terms of AUC for the co-expression prediction based on standard and scale-aware topological measure of chromatin interaction networks. A gene-pair is labeled co-expressed (i.e. positive class) or not co-expressed (i.e. negative class) if their correlation is above or below the median (i.e. 50^*th*^-percentile) of all correlations across all chromosomes, respectively. B) Classification performance using Hi-C interaction above the median. Classification performance in terms of AUC for the co-expression prediction based on standard and scale-aware topological measure of chromatin interaction networks. Each box represents the classifier performance for all mouse chromosomes. Multi-resolution refers to concatenated feature set of topological measures obtained from CINs at a Hi-C resolution of 40, 80, 120, 160, and 200kb. The performance of the classifier (RNN with 800 hidden nodes) is determined using 10-fold cross validation. In each box, the horizontal line represents the median. The thick vertical line represents the interval of *q*_1_ = 25^*th*^ and *q*_3_ = 75^*th*^ percentiles. The thin vertical line represents the interval of *q*_3_ + 1.5(*q*_3_ − *q*_1_) and *q*_1_ − 1.5(*q*_3_ − *q*_1_).
(TIF)

S7 Fig Classification performance using average-based normalization of Hi-C matrices. Classification performance in terms of AUC for the co-expression prediction based on standard and scale-aware topological measure of chromatin interaction networks which are built based on Hi-C matrices after average-based normalization. Each box encompasses the classifier performance for all mouse chromosomes. Multi-resolution refers to concatenated feature set of topological measures obtained from CINs at Hi-C resolution of 40, 80, 120, 160, and 200*kb*. The performance of the classifier (RNN with 800 hidden nodes) is determined using 10-fold cross validation. In each box, the horizontal line represents the median. The thick vertical line represents the interval of *q*_1_ = 25^*th*^ and *q*_3_ = 75^*th*^ percentiles. The thin vertical line represents the interval of *q*_3_ + 1.5(*q*_3_ − *q*_1_) and *q*_1_ − 1.5(*q*_3_ − *q*_1_).
(TIF)

S8 Fig Classification performance using the bin-based CIN. Classification performance in terms of AUC for the co-expression prediction based on standard and scale-aware topological measure of the chromatin interaction network which is built based on the all genomic loci (i.e. non-overlapping bins with size of 200kb) within a chromosome. Each box represents the classifier performance for all mouse chromosomes. The performance of the classifier (RNN with 800 hidden nodes) is determined using 10-fold cross validation. In each box, the horizontal line represents the median. The thick vertical line represents the interval of *q*_1_ = 25^*th*^ and *q*_3_ = 75^*th*^ percentiles. The thin vertical line represents the interval of *q*_3_ + 1.5(*q*_3_ − *q*_1_) and *q*_1_ − 1.5(*q*_3_ − *q*_1_).
(TIF)

S9 Fig Classification performance using the individual standard topological measures. Classification performance in terms of AUC using the individual standard topological measures (8 measures) across a range of Hi-C resolutions (5 resolutions). The performance of the classifier (RNN with 100 hidden nodes) was determined using 5-fold cross validation.
(TIF)

S10 Fig Classification performance using the individual scale-aware topological measures. Classification performance in terms of AUC using the individual scale-aware topological measures (8 measures) across a range of Hi-C resolutions (5 resolutions) and 10 scales (400 measures) for Chromosome 3, 10 and 16. The performance of the classifier (RNN with 100 hidden nodes) is determined using 5-fold cross validation.
(TIF)

S11 Fig Classification performance using the forward feature selection method. Classification performance in terms of AUC for the co-expression prediction based on all scale-aware topological measure of chromatin interaction networks and selected measures using the forward feature selection method. Each box encompasses the classifier performance for all mouse chromosomes. Multi-resolution refers to concatenated feature set of topological measures obtained from CINs at Hi-C resolution of 40, 80, 120, 160, and 200*kb*. The performance of the classifier (RNN with 100 hidden nodes) is determined using 5-fold cross validation. In each box, the horizontal line represents the median. The thick vertical line represents the interval of *q*_1_ = 25^*th*^ and *q*_3_ = 75^*th*^ percentiles. The thin vertical line represents the interval of *q*_3_ + 1.5(*q*_3_ − *q*_1_) and *q*_1_ − 1.5(*q*_3_ − *q*_1_).
(TIF)

S12 Fig 2D maps of multi-resolution standard topological measures. 2D maps of multi-resolution standard topological measures (80 measures) for all mouse chromosomes. Each point in the map indicates the topological properties of the interaction profile between a gene-pair. Red and blue indicate strong and low co-expression between corresponding gene-pairs, respectively.
(TIF)

S13 Fig 2D maps of multi-resolution scale-aware topological measures. 2D maps of multi-resolution scale-aware topological measures (800 measures) for all mouse chromosomes. Each point in the map indicates the topological properties of the interaction profile between a gene-pair. Red and blue indicate strong and low co-expression between corresponding gene-pairs, respectively.
(TIF)

S14 Fig 2D maps of standard and selected STM features. 2D maps of standard (80 measures) and selected STM features (206 STMs, obtained using the feature selection procedure) for the 40*kb* (A) and 200*kb* (B and C) CIN of Chromosome 16. Each point in the map indicates the topological properties of the interaction profile between a gene-pair. Points are colored based on the A) clustering coefficient at 40kb resolution B) clustering coefficient at 200*kb* resolution and C) Jaccard index at 200*kb* resolution measures across the scales.
(TIF)

S15 Fig ROC curve of the co-expression prediction based on the CIN of Chromosome 16. The area under each curve shows the performance of classification for different setting of the CIN.
(TIF)
