# Supplementary figures and images for: Hi-C Chromatin Interaction Networks Predict Co-expression in the Mouse Cortex
Source: PLoS Comput Biol. 2015 May 12;11(5):e1004221. doi: 10.1371/journal.pcbi.1004221 (PMC4429121; doi:10.1371/journal.pcbi.1004221)

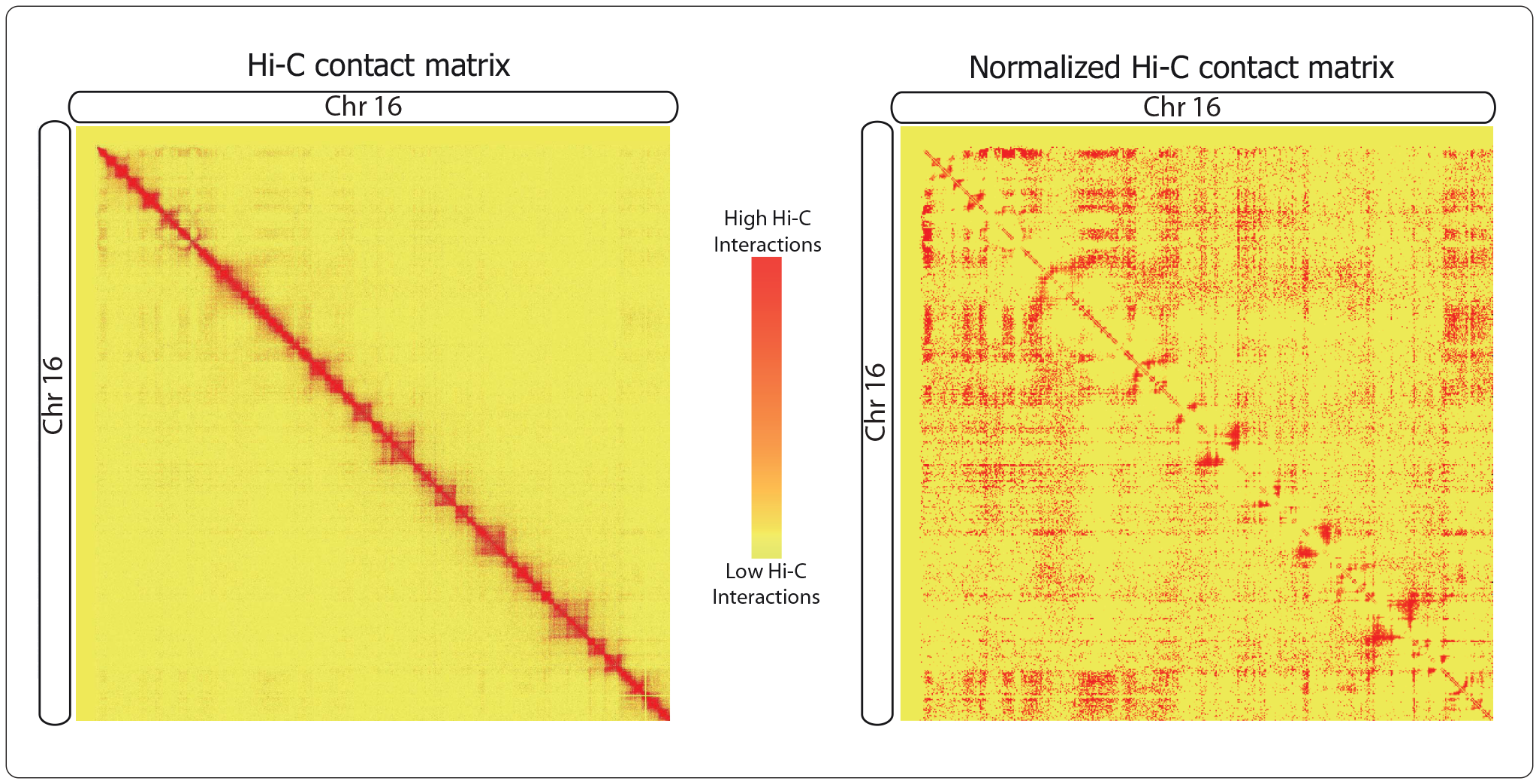

Supplement: S1 Fig — (A) Hi-C contact matrix of Chromosome 16. (B) Rank-normalized Hi-C contact matrix. The genomic distance bias in the Hi-C contact matrix is eliminated by using a rank-based normalization (described in the main text). (TIF) [file pcbi.1004221.s003.tif]

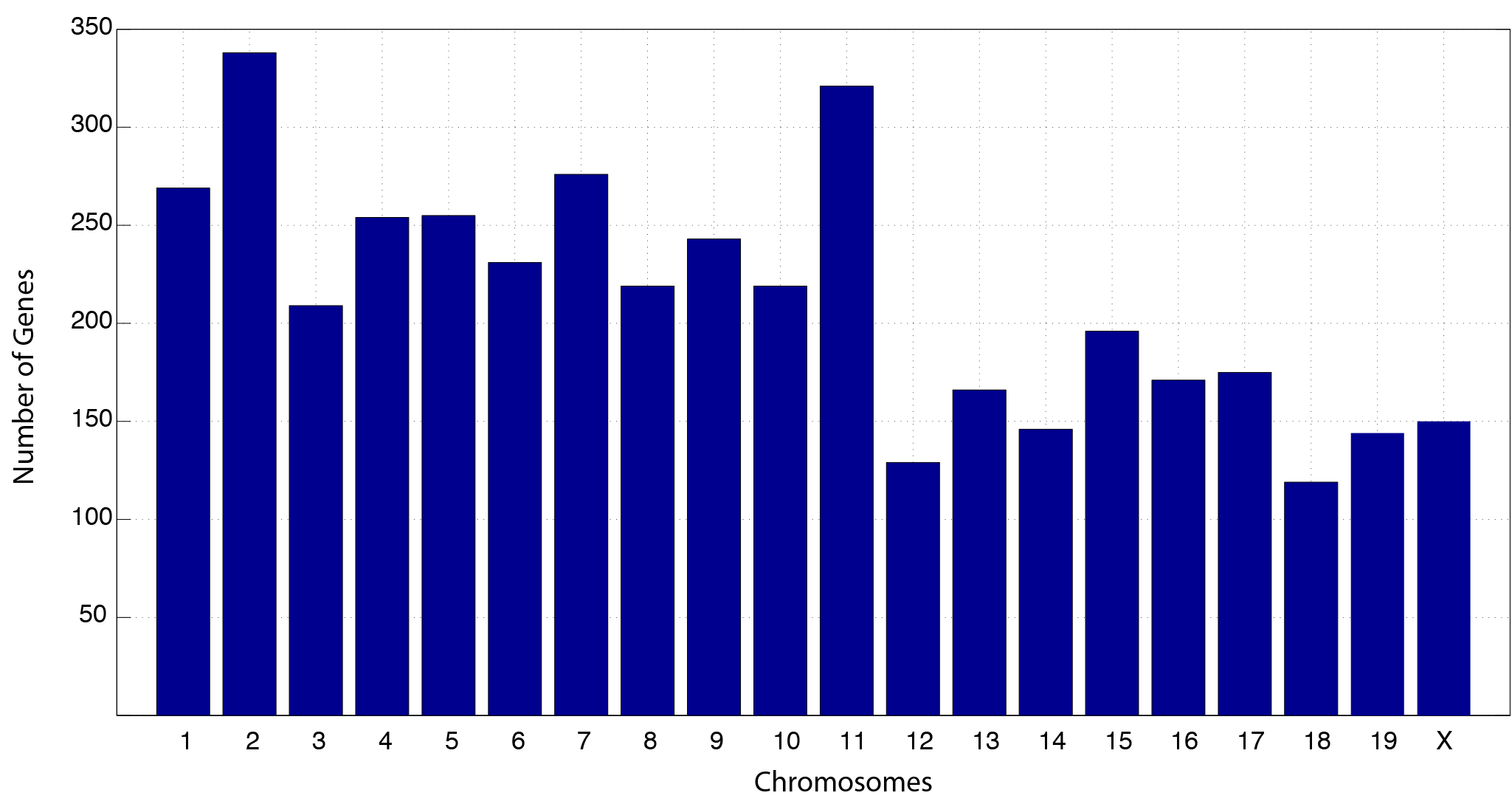

Supplement: S2 Fig — (TIF) [file pcbi.1004221.s004.tif]

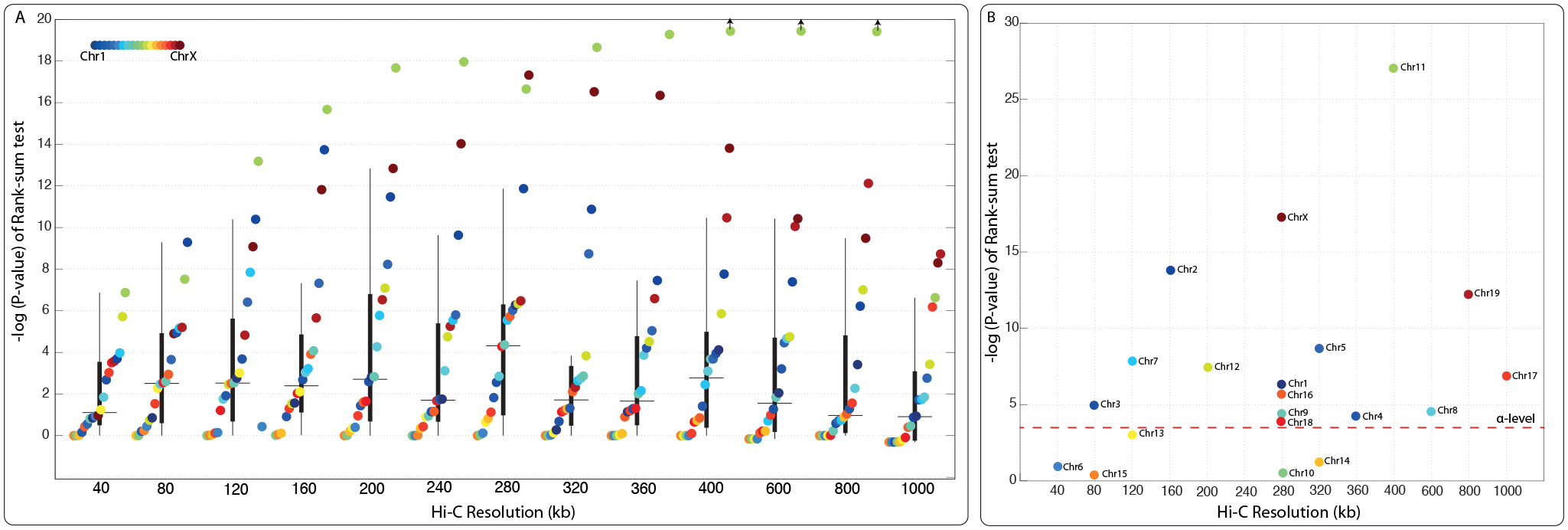

Supplement: S3 Fig — (A) Assessment of the enrichment of Hi-C interactions between strongly co-expressed gene-pairs compared to gene-pairs with no co-expression across different Hi-C resolutions. The y-axis indicates −log 10(p − value) of the one-tailed Wilcoxon rank-sum test used for the enrichment analysis. Hi-C interactions were mapped to genes using the TSS-mapping method. (B) Overview of the Hi-C resolution at which Hi-C interactions are most significantly associated with co-expressed gene-pairs for each chromosome. In each box, the horizontal line represents the median. The thick vertical line represents the interval of q 1 = 25th and q 3 = 75th percentiles. The thin vertical line represents the interval of q 3 + 1.5(q 3 − q 1) and q 1 − 1.5(q 3 − q 1). (TIF) [file pcbi.1004221.s005.tif]

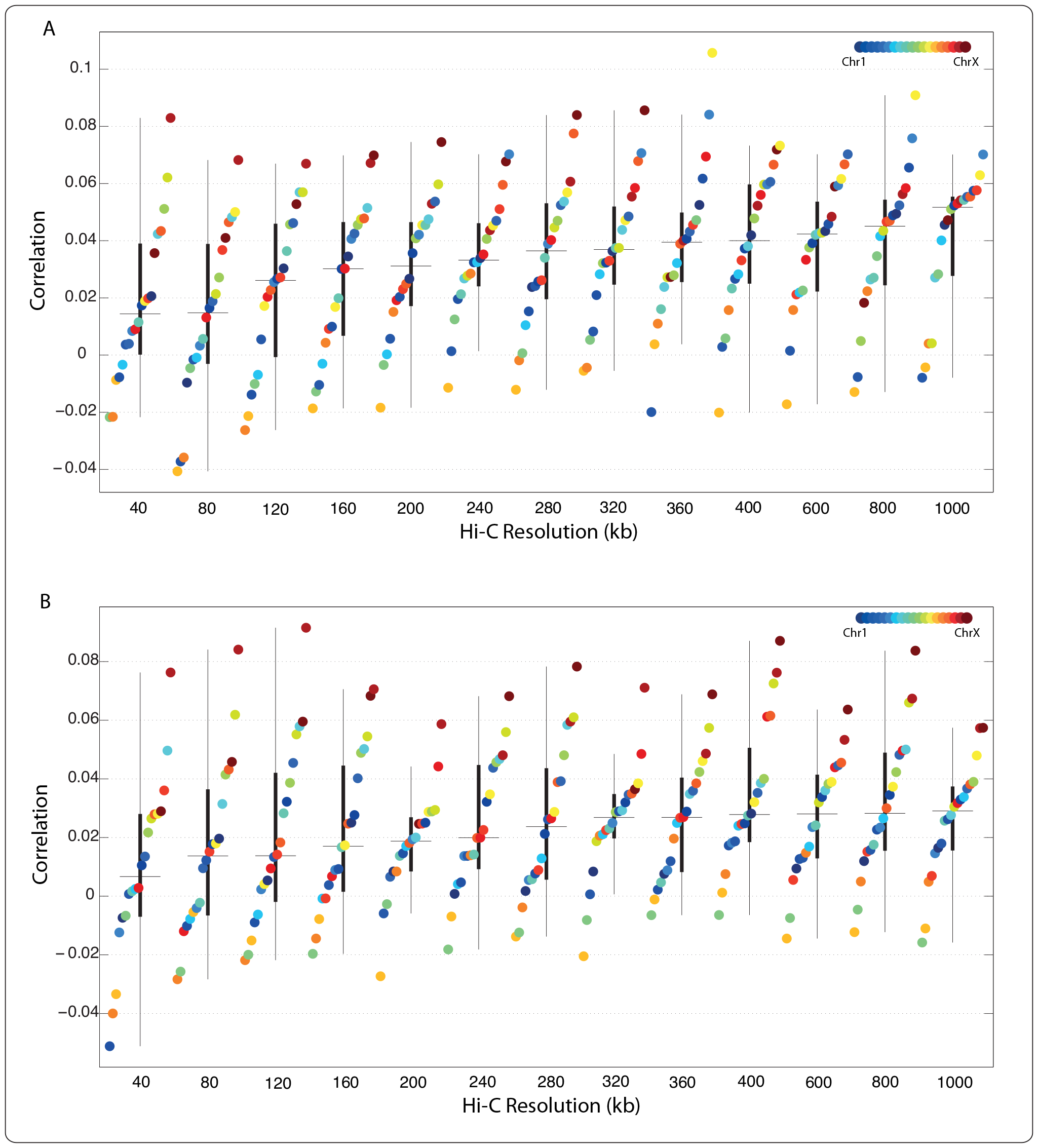

Supplement: S4 Fig — The Pearson’s correlation coefficient between the Hi-C matrix and co-expression matrix of all gene-pairs in each chromosome. Each box represents the correlations for all mouse chromosomes at a specific Hi-C resolution. Hi-C interactions between genes were determined using (A) the MAX-mapping method and (B) the TSS-mapping method. In each box, the horizontal line represents the median. The thick vertical line represents the interval of q 1 = 25th and q 3 = 75th percentiles. The thin vertical line represents the interval of q 3 + 1.5(q 3 − q 1) and q 1 − 1.5(q 3 − q 1). (TIF) [file pcbi.1004221.s006.tif]

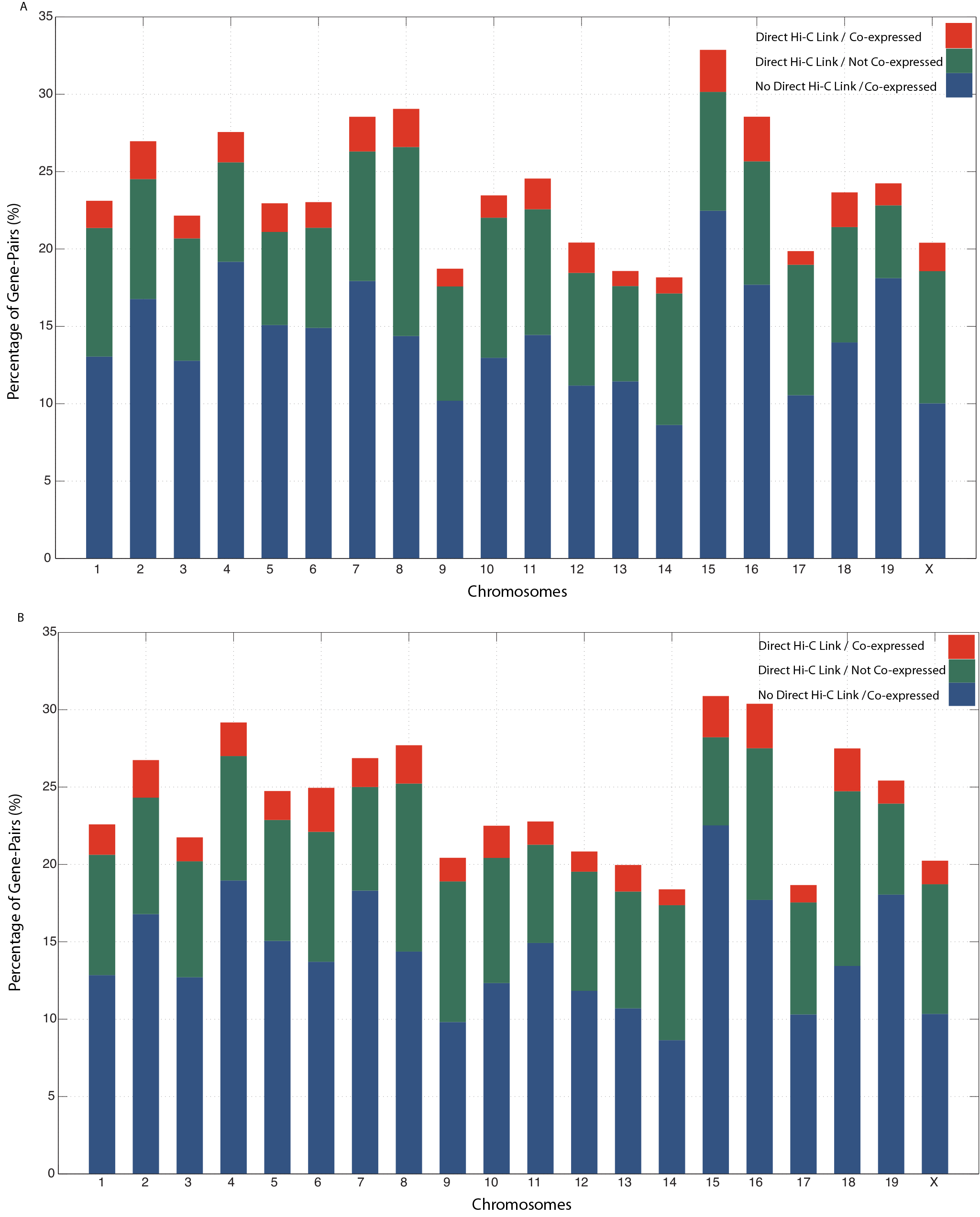

Supplement: S5 Fig — Percentage of interacting genes that co-express, the percentage of interacting genes that do not co-express, and the percentage of non-interacting genes that co-express per chromosome in CINs at A) 200kb, B) 40kb resolution. The percentage of gene-pairs with either a Hi-C link or co-expressed is about 22% (average across all chromosomes). Additionally, we also observed the percentage of co-expressed gene-pairs with a Hi-C link is very low per chromosome (2% average across all chromosomes). (TIF) [file pcbi.1004221.s007.tif]

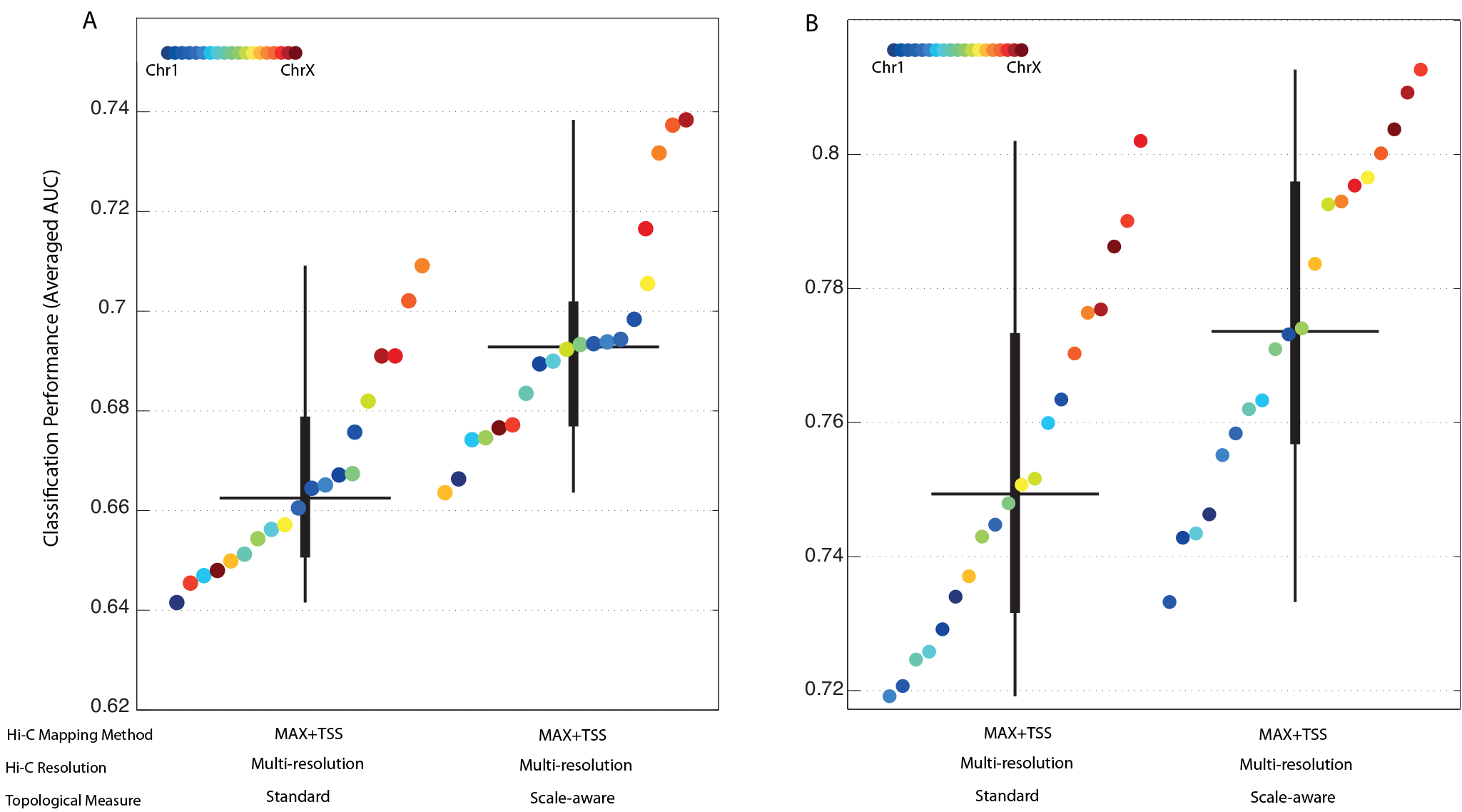

Supplement: S6 Fig — A) Classification performance using all co-expression links. Classification performance in terms of AUC for the co-expression prediction based on standard and scale-aware topological measure of chromatin interaction networks. A gene-pair is labeled co-expressed (i.e. positive class) or not co-expressed (i.e. negative class) if their correlation is above or below the median (i.e. 50th-percentile) of all correlations across all chromosomes, respectively. B) Classification performance using Hi-C interaction above the median. Classification performance in terms of AUC for the co-expression prediction based on standard and scale-aware topological measure of chromatin interaction networks. Each box represents the classifier performance for all mouse chromosomes. Multi-resolution refers to concatenated feature set of topological measures obtained from CINs at a Hi-C resolution of 40, 80, 120, 160, and 200kb. The performance of the classifier (RNN with 800 hidden nodes) is determined using 10-fold cross validation. In each box, the horizontal line represents the median. The thick vertical line represents the interval of q 1 = 25th and q 3 = 75th percentiles. The thin vertical line represents the interval of q 3 + 1.5(q 3 − q 1) and q 1 − 1.5(q 3 − q 1). (TIF) [file pcbi.1004221.s008.tif]

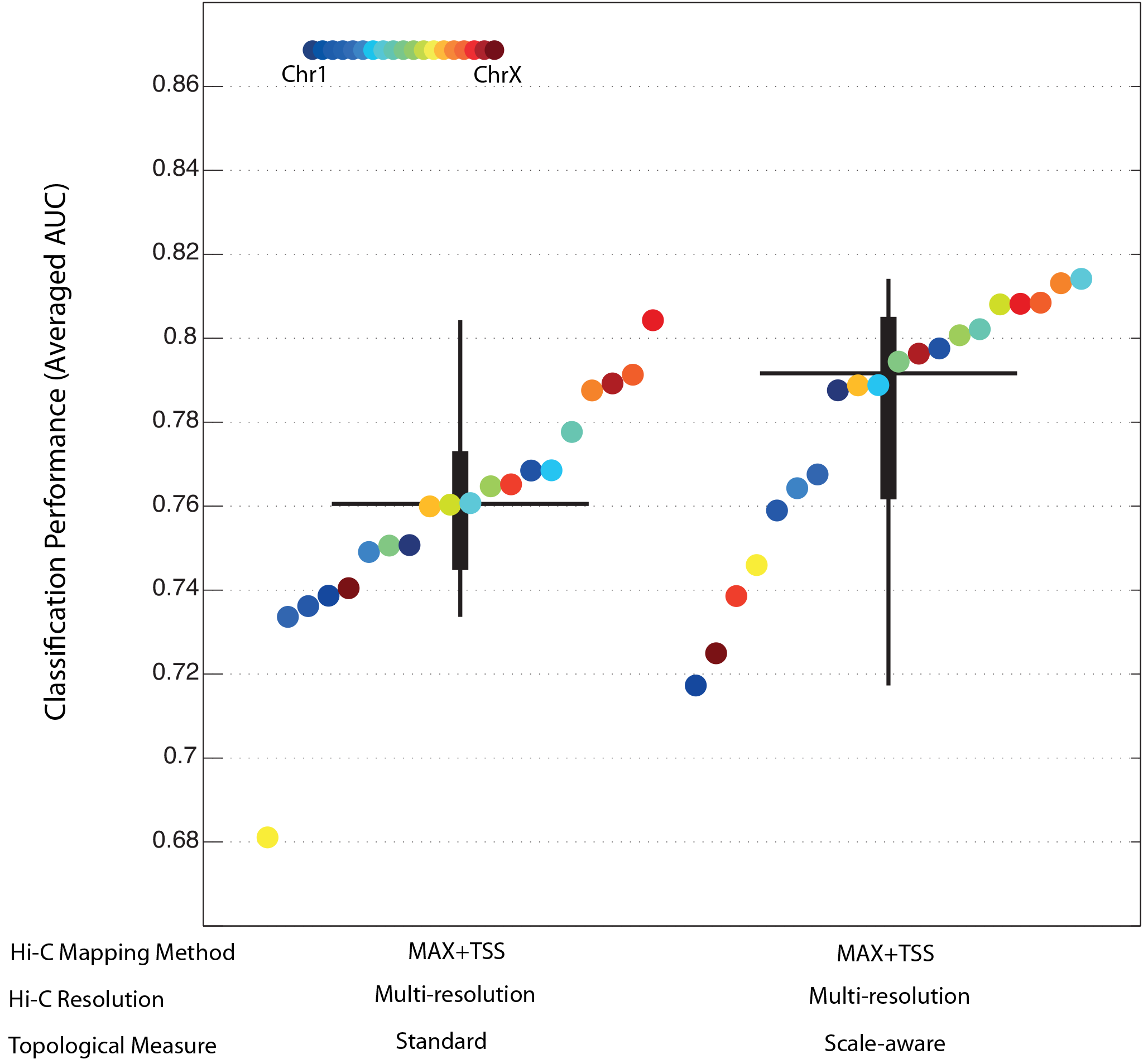

Supplement: S7 Fig — Classification performance in terms of AUC for the co-expression prediction based on standard and scale-aware topological measure of chromatin interaction networks which are built based on Hi-C matrices after average-based normalization. Each box encompasses the classifier performance for all mouse chromosomes. Multi-resolution refers to concatenated feature set of topological measures obtained from CINs at Hi-C resolution of 40, 80, 120, 160, and 200kb. The performance of the classifier (RNN with 800 hidden nodes) is determined using 10-fold cross validation. In each box, the horizontal line represents the median. The thick vertical line represents the interval of q 1 = 25th and q 3 = 75th percentiles. The thin vertical line represents the interval of q 3 + 1.5(q 3 − q 1) and q 1 − 1.5(q 3 − q 1). (TIF) [file pcbi.1004221.s009.tif]

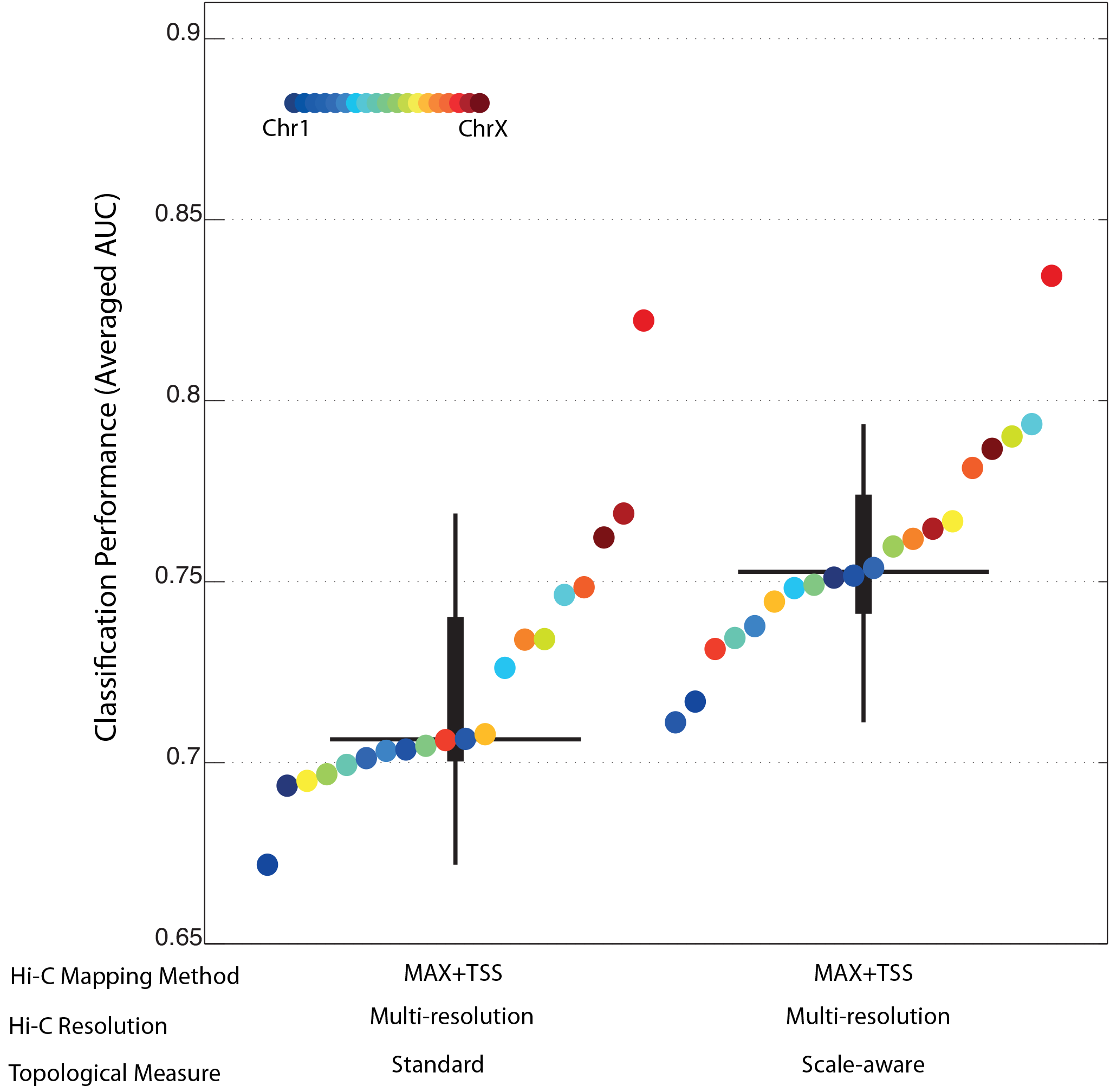

Supplement: S8 Fig — Classification performance in terms of AUC for the co-expression prediction based on standard and scale-aware topological measure of the chromatin interaction network which is built based on the all genomic loci (i.e. non-overlapping bins with size of 200kb) within a chromosome. Each box represents the classifier performance for all mouse chromosomes. The performance of the classifier (RNN with 800 hidden nodes) is determined using 10-fold cross validation. In each box, the horizontal line represents the median. The thick vertical line represents the interval of q 1 = 25th and q 3 = 75th percentiles. The thin vertical line represents the interval of q 3 + 1.5(q 3 − q 1) and q 1 − 1.5(q 3 − q 1). (TIF) [file pcbi.1004221.s010.tif]

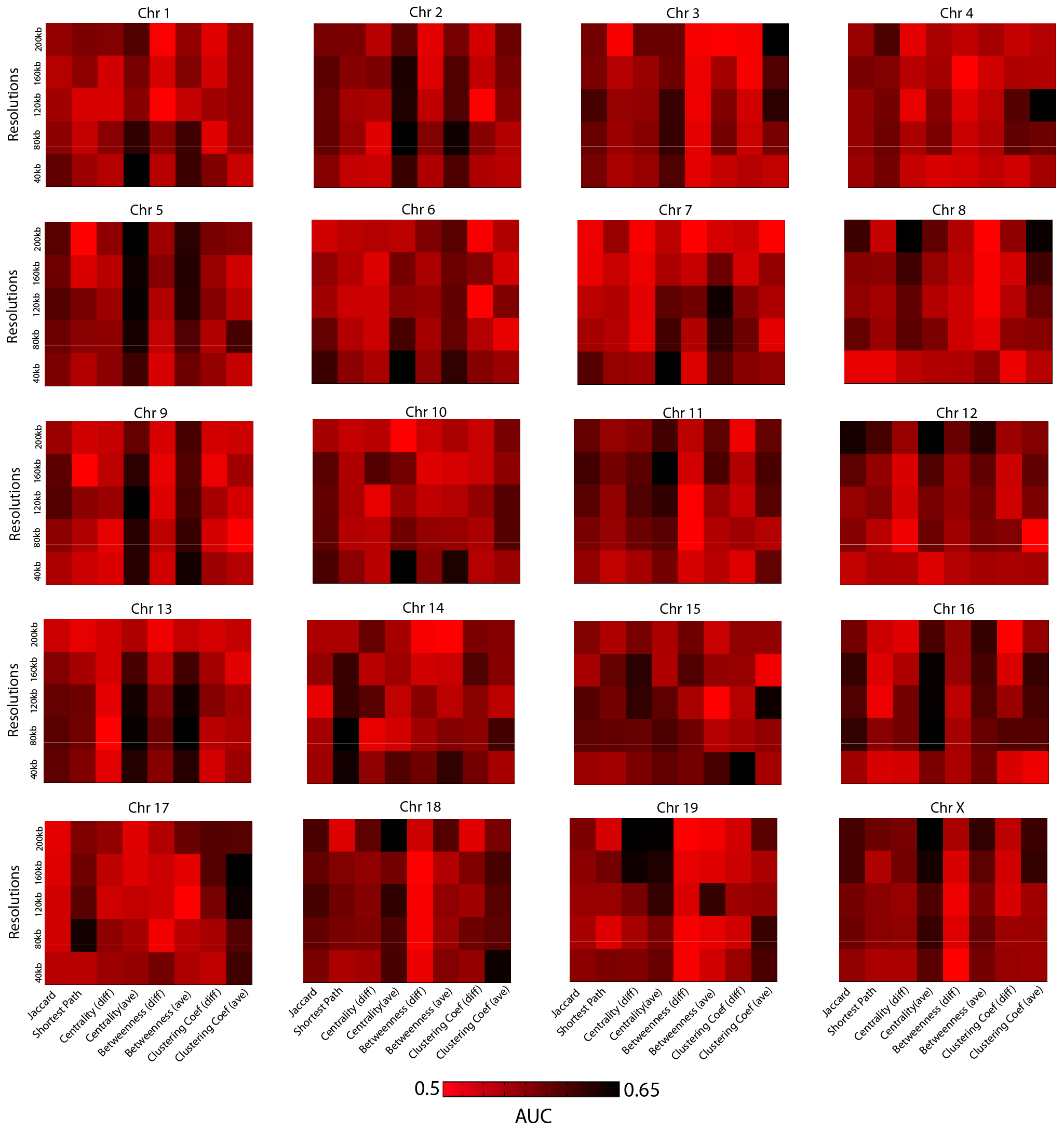

Supplement: S9 Fig — Classification performance in terms of AUC using the individual standard topological measures (8 measures) across a range of Hi-C resolutions (5 resolutions). The performance of the classifier (RNN with 100 hidden nodes) was determined using 5-fold cross validation. (TIF) [file pcbi.1004221.s011.tif]

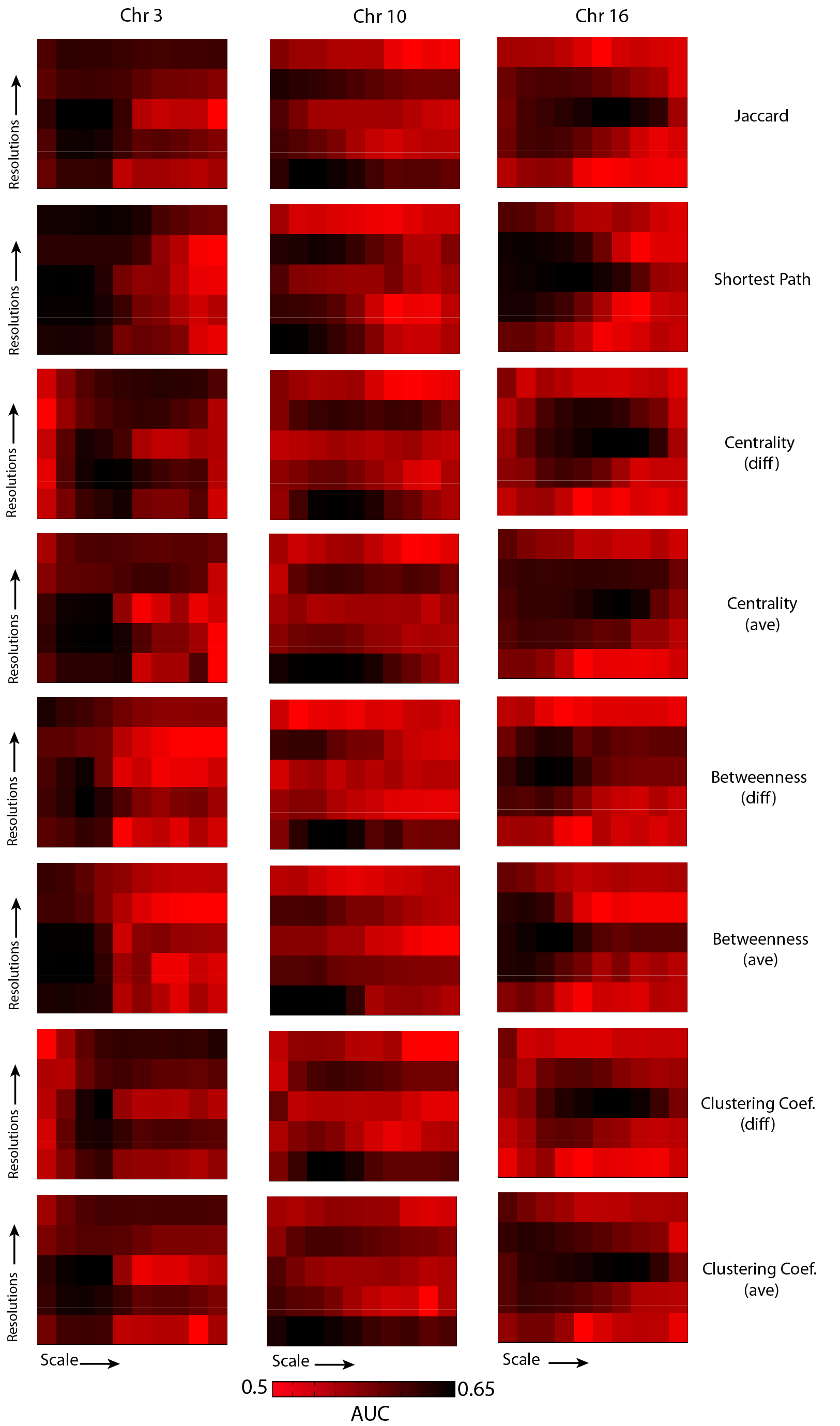

Supplement: S10 Fig — Classification performance in terms of AUC using the individual scale-aware topological measures (8 measures) across a range of Hi-C resolutions (5 resolutions) and 10 scales (400 measures) for Chromosome 3, 10 and 16. The performance of the classifier (RNN with 100 hidden nodes) is determined using 5-fold cross validation. (TIF) [file pcbi.1004221.s012.tif]

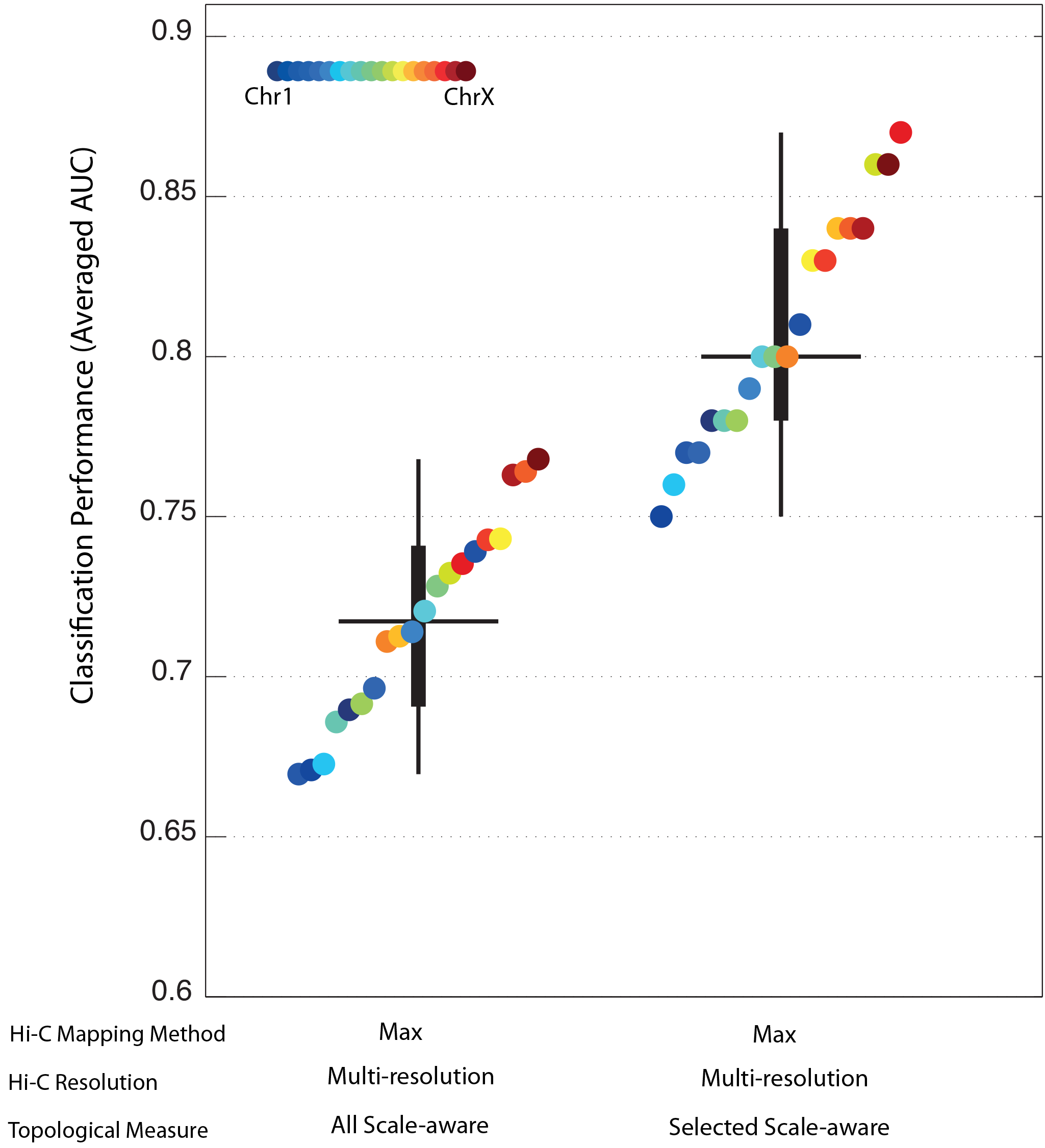

Supplement: S11 Fig — Classification performance in terms of AUC for the co-expression prediction based on all scale-aware topological measure of chromatin interaction networks and selected measures using the forward feature selection method. Each box encompasses the classifier performance for all mouse chromosomes. Multi-resolution refers to concatenated feature set of topological measures obtained from CINs at Hi-C resolution of 40, 80, 120, 160, and 200kb. The performance of the classifier (RNN with 100 hidden nodes) is determined using 5-fold cross validation. In each box, the horizontal line represents the median. The thick vertical line represents the interval of q 1 = 25th and q 3 = 75th percentiles. The thin vertical line represents the interval of q 3 + 1.5(q 3 − q 1) and q 1 − 1.5(q 3 − q 1). (TIF) [file pcbi.1004221.s013.tif]

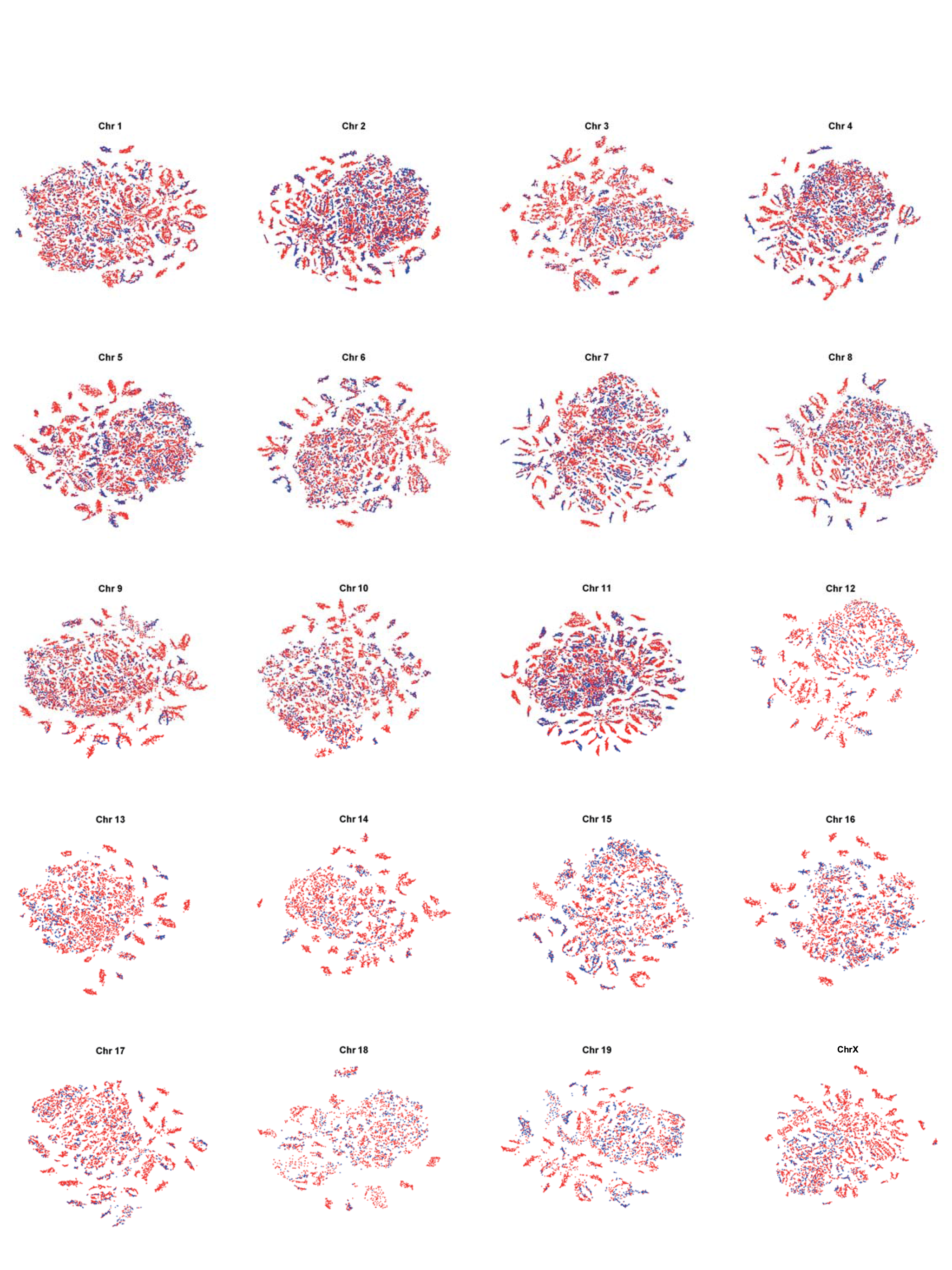

Supplement: S12 Fig — 2D maps of multi-resolution standard topological measures (80 measures) for all mouse chromosomes. Each point in the map indicates the topological properties of the interaction profile between a gene-pair. Red and blue indicate strong and low co-expression between corresponding gene-pairs, respectively. (TIF) [file pcbi.1004221.s014.tif]

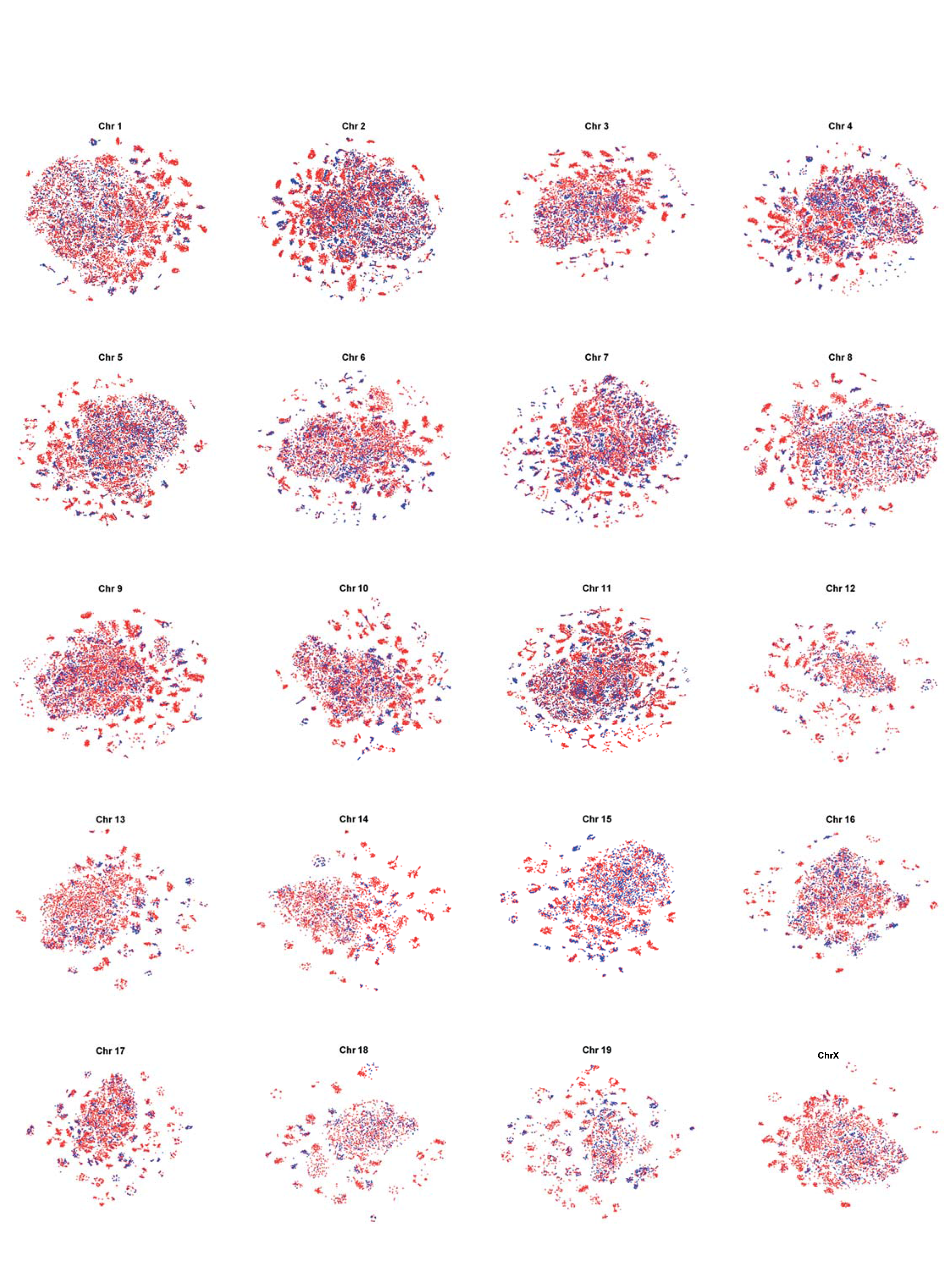

Supplement: S13 Fig — 2D maps of multi-resolution scale-aware topological measures (800 measures) for all mouse chromosomes. Each point in the map indicates the topological properties of the interaction profile between a gene-pair. Red and blue indicate strong and low co-expression between corresponding gene-pairs, respectively. (TIF) [file pcbi.1004221.s015.tif]

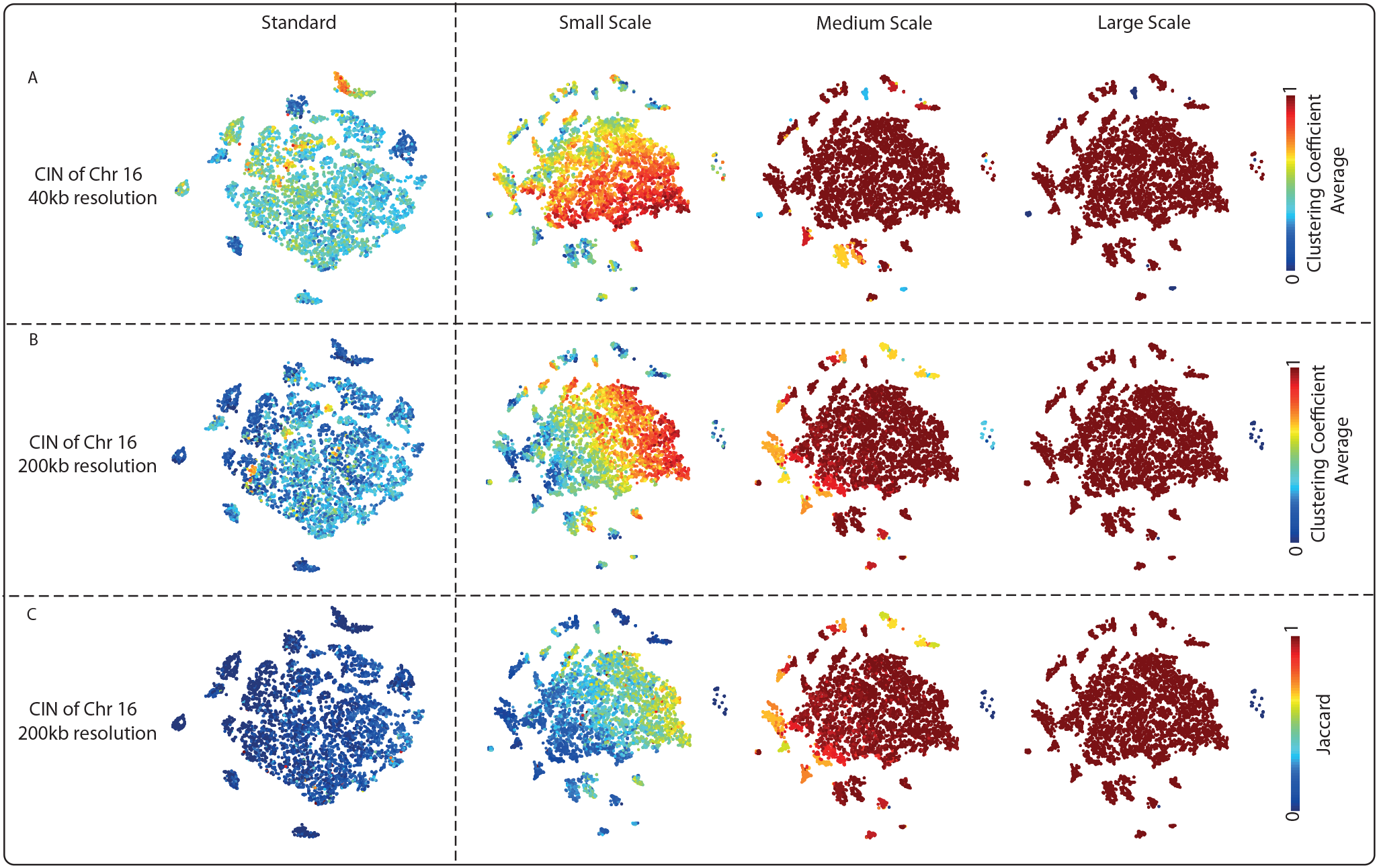

Supplement: S14 Fig — 2D maps of standard (80 measures) and selected STM features (206 STMs, obtained using the feature selection procedure) for the 40kb (A) and 200kb (B and C) CIN of Chromosome 16. Each point in the map indicates the topological properties of the interaction profile between a gene-pair. Points are colored based on the A) clustering coefficient at 40kb resolution B) clustering coefficient at 200kb resolution and C) Jaccard index at 200kb resolution measures across the scales. (TIF) [file pcbi.1004221.s016.tif]

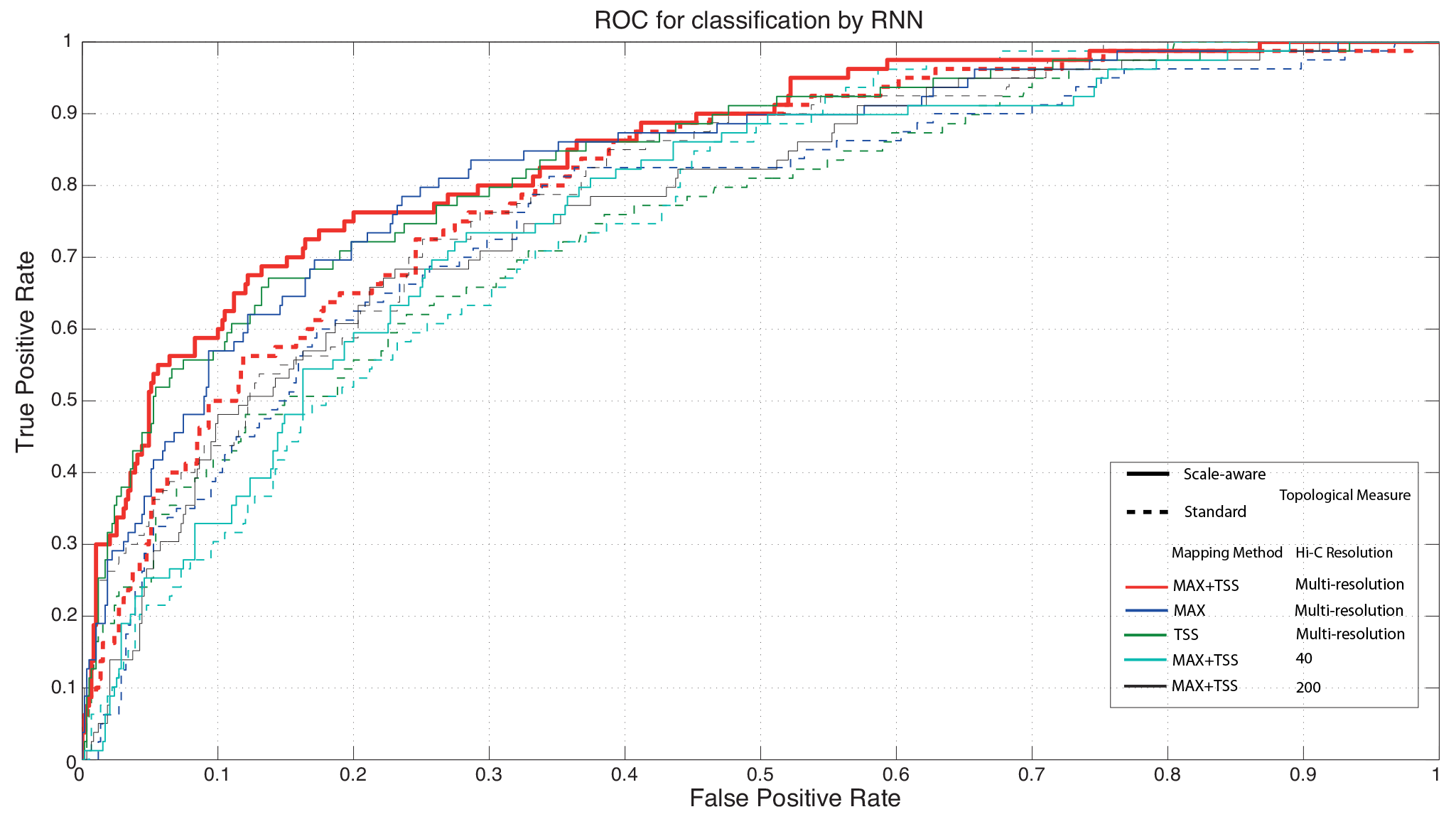

Supplement: S15 Fig — The area under each curve shows the performance of classification for different setting of the CIN. (TIF) [file pcbi.1004221.s017.tif]
